# Validation of a small molecule inhibitor of PDE6D-RAS interaction with favorable anti-leukemic effects

**DOI:** 10.1038/s41408-022-00663-z

**Published:** 2022-04-14

**Authors:** Sara Canovas Nunes, Serena De Vita, Andrew Anighoro, François Autelitano, Edward Beaumont, Pamela Klingbeil, Meaghan McGuinness, Beatrice Duvert, Chad Harris, Lu Yang, Sheela Pangeni Pokharel, Chun-Wei Chen, Monika Ermann, David A. Williams, Haiming Xu

**Affiliations:** 1 Division of Hematology/Oncology, Boston Children’s Hospital, Harvard Medical School, Boston, MA USA; 2 Evotec SE, Manfred Eigen Campus, Hamburg, Germany; 3 Department of Systems Biology, Beckman Research Institute of City of Hope, Monrovia, CA USA; 4 Department of Pediatric Oncology, Dana-Farber Cancer Institute, Boston, MA USA; 5 Harvard Stem Cell Institute, Harvard University, Boston, MA USA; 6 Harvard Initiative for RNA Medicine, Harvard Medical School, Boston, MA USA

**Keywords:** Acute lymphocytic leukaemia, Acute myeloid leukaemia, Drug development, Targeted therapies

## Abstract

RAS mutations prevalent in high-risk leukemia have been linked to relapse and chemotherapy resistance. Efforts to directly target RAS proteins have been largely unsuccessful. However, since RAS-mediated transformation is dependent on signaling through the RAS-related C3 botulinum toxin substrate (RAC) small GTPase, we hypothesized that targeting RAC may be an effective therapeutic approach in RAS mutated tumors. Here we describe multiple small molecules capable of inhibiting RAC activation in acute lymphoblastic leukemia cell lines. One of these, DW0254, also demonstrates promising anti-leukemic activity in RAS-mutated cells. Using chemical proteomics and biophysical methods, we identified the hydrophobic pocket of phosphodiester 6 subunit delta (PDE6D), a known RAS chaperone, as a target for this compound. Inhibition of RAS localization to the plasma membrane upon DW0254 treatment is associated with RAC inhibition through a phosphatidylinositol-3-kinase/AKT-dependent mechanism. Our findings provide new insights into the importance of PDE6D-mediated transport for RAS-dependent RAC activation and leukemic cell survival.

## Introduction

Guanosine triphosphatases (GTPases) are small G proteins that play key roles in hematopoietic cells in a variety of cellular processes, including proliferation, apoptosis, cell migration, and cytoskeleton rearrangements [1, 2]. Activating mutations in RAS GTPase isoforms have been linked to numerous types of human cancers, including myeloid and lymphoid malignancies [3–6]. NRAS/KRAS mutations have been found in 20–25% of patients with acute myeloid leukemia (AML) [5], 25–30% of patients with juvenile myelomonocytic leukemia (JMML) [7], and 15% of pediatric patients with B- or T-lineage acute lymphoblastic leukemia (ALL) [8, 9]. Specifically, RAS mutations are highly prevalent in relapsed high-risk ALL after combination chemotherapy, and the activation of RAS signaling has been shown to act as the driver of both *de novo* and relapsed, chemotherapy-resistant disease [10, 11]. The various attempts to develop drugs that directly target mutant RAS proteins have been largely unsuccessful, and to this day, only specific KRAS G12C inhibitors have been developed with evidence of clinical activity in solid tumors [12, 13]. However, this specific mutation is usually not found in relapsed acute leukemia patients [11].

Since, in some model systems, RAS-related C3 botulinum toxin substrate (RAC) GTPase is required for full RAS transformation [14] and leukemia cell survival [15, 16], we and others have focused on inhibiting its activity to indirectly target RAS signaling [17]. Here we report the identification of a compound DW0069 and the development of two derivatives, DW0254 and DW0441, which demonstrated dose-dependent RAC inhibition, arrest of proliferation and induced apoptosis in human leukemic cell lines. We found that these compounds bind the hydrophobic pocket of phosphodiester 6 subunit delta (PDE6D), a RAS chaperone protein. Directed mutation of this pocket led to compound resistance, directly implicating molecule binding to PDE6D to cell growth inhibition. We further showed that treatment with DW0254 disrupts the interaction between PDE6D and RAS, disturbing RAS subcellular localization. Moreover, the dose-dependent decrease in RAC activation downstream of phosphatidylinositol 3-kinase/protein kinase B (PI3K/AKT) provides a biochemical link between RAS and RAC in leukemia cells. In summary, our study provides evidence that PDE6D-dependent RAS trafficking with downstream activation of PI3K/AKT and RAC constitutes a novel potential therapeutic target in high risk leukemias.

## Results

### Discovery of small molecules with antileukemic activity and identification of their direct binding target PDE6D

**Fig. 1:**
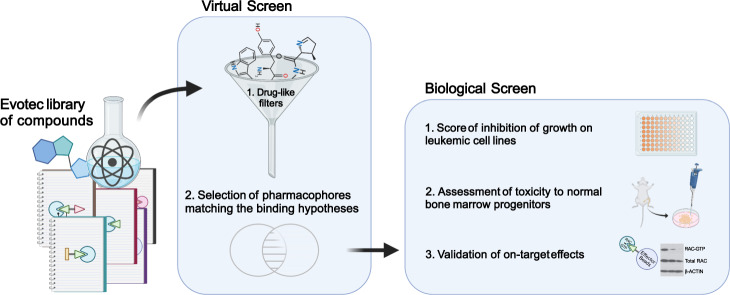
Compound screen for Rac inhibitors. A virtual screen of Evotec’s library of commercially available compounds was performed that included an initial filtration for drug-like properties, followed by a preselection against shape-based pharmacophore of published RAC inhibitors. Next, a biological screen was carried out consisting of: (i) scoring for inhibition of growth on leukemic cell lines of 107 selected compounds, (ii) assessment of toxicity to normal bone marrow progenitors of compounds that showed good anti-leukemic activity, and (iii) validating on-target effects by RAC PBD pull down of non-toxic compounds. Created with BioRender.com.

**Fig. 2:**
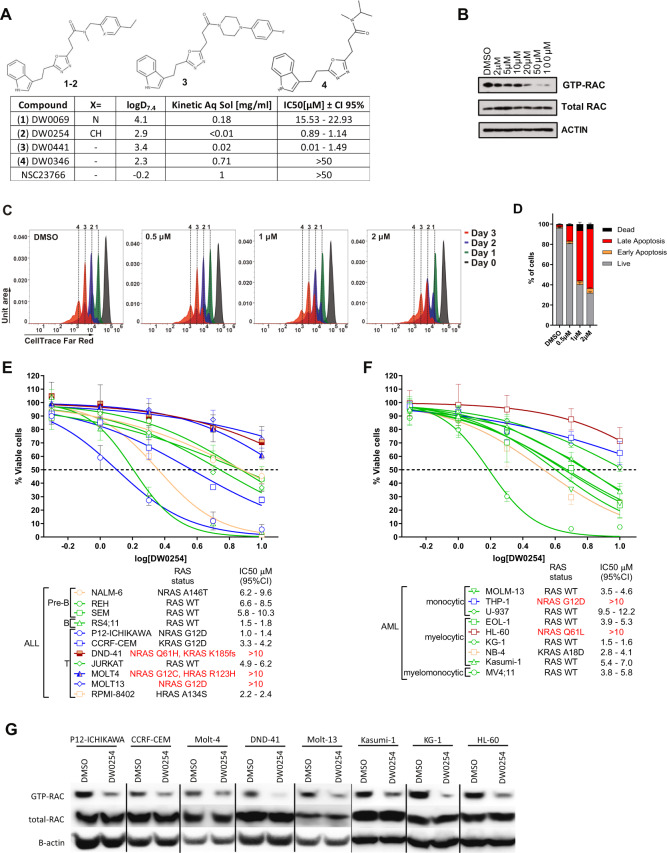
Compound DW0254 inhibits RAC activation and shows anti-leukemic activity in vitro in leukemia cell lines. **A** Chemical structure, physicochemical properties, and biological activities cell line for compounds 1–4 and NSC23766 (structure not shown), a known inhibitor of RAC. IC_50_ values represent the dose at which 50% cell viability was achieved on P12-ICHIKAWA cells. **B** GTP-RAC activity inhibition in P12-ICHIKAWA cells treated with different doses of DW0254 for 3 h. GST pulldown assays were conducted by incubating lysates with PAK1-PBD beads. Cell lysates to detect total RAC and proteins eluted from the PAK1-PBD beads to detect GTP-RAC were subjected to Western blotting using anti-RAC (610651, BD Transduction laboratories, San Jose, CA) and anti-beta ACTIN (A5441, Sigma-Aldrich) antibodies. Data are representative of three individual experiments. **C** Representative peaks of Far Red CellTrace staining of P12-ICHIKAWA cells treated with different doses of DW0254 and examined by FACS on three consecutive days. Peaks 1–4 represent the number of times the cells in each peak have divided; data shown from one of the three independent experiments. **D** Bar graph showing percentage of apoptosis by AnnV/PI staining of P12-ICHIKAWA cells treated for 3 days with different doses of DW0254, data represent mean ± SD of two independent experiments with *n* = 3 samples for each condition. Live: AnnV^−^/PI^−^; Early apoptosis: AnnV^+^/PI^−^; Late apoptosis: AnnV^+^/PI^+^; and Dead: AnnV^−^/PI^+^. **E** Drug dosage curve showing live cell viability assay after 3 days of DW0254 treatment of human ALL and **F** AML cell lines with diverse backgrounds and RAS status as described, *n* = 4 at each dosage, data show mean ± SD, one of three individual experiments showing the consistent results. Color code: WT RAS green, G12 mutant RAS blue, Q61 mutant RAS burgundy, other RAS mutations yellow. **G** GTP-RAC activity inhibition in a panel of T-ALL and AML cell lines treated with 50 µM DW0254 for 1 h.

Our initial screen for a RAC inhibitor depicted in Fig. 1 lead to the identification of compound DW0069, and further medicinal chemistry efforts yielded the closely related compounds DW0254 and DW0441 (1, 2, and 3 respectively in Fig. 2A) [18]. These early leads had suboptimal to satisfactory physiochemical properties although all showed improved biological activity on leukemia cells when compared to the tool RAC inhibitor NSC23766 [19] which showed cellular activities in the ~40–80 µM range ([20] and Fig. 2A). In contrast, DW0346 analogue with an aliphatic amide substitution (4 in Fig. 2A) showed a significant reduction in inhibitory activity on leukemic cells and was used as a negative control in subsequent target validation experiments. DW0254 was further profiled as it offered the best compromise between lipophilicity, solubility, and potent biological activity. Treatment of P12-ICHIKAWA cells, caused a dose-dependent inhibition of RAC activation (Fig. 2B), decrease in cell proliferation (Fig. 2C) and increase in apoptosis (Fig. 2D). DW0069 and DW0441 also affected cell growth, apoptosis, and RAC activation (Supplemental Fig. 1A–E). DW0254 antileukemic activity was tested on a large panel of ALL and AML cell lines that exhibited varying levels of sensitivity to DW0254 (Fig. 2E, F). Seventy-five percent were considered responsive with a mean IC_50_ between 1 and 10 µM. Both sensitive and resistant cell lines exhibited decreased GTP-RAC levels upon DW0254 treatment (Fig. 2G).

**Fig. 3:**
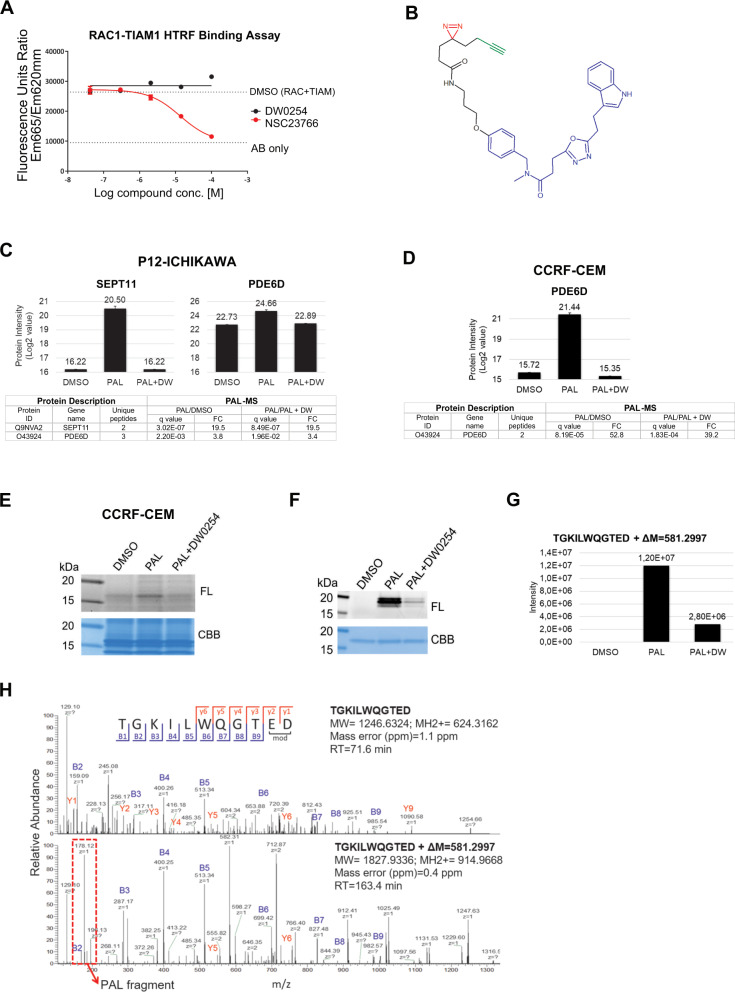
Identification of the DW0254 molecular target PDE6D by photoaffinity labeling mass spectrometry (PALMS). **A** Compound titration in the RAC1-TIAM1 homogeneous time-resolved fluorescence assay (HTRF) assay showing competition with increasing concentrations of test compounds (either NSC23766 or DW0254) on *X*-axis and fluorescence emission (*Y*-axis). **B** Chemical structure of DW0254-photoprobe PAL. The DW0254 warhead is colored in blue, the photoreactive diazirine group in red, and the alkyne clickable group in green. **C** top: MS signal intensity of protein target hits of DW0254 in the pulldown samples of P12-ICHIKAWA cells. Histogram plots represent quantitative determination of PDE6D and SEPT11 MS signal intensity in the different pulldown samples (DMSO, PAL alone, and PAL in combination with a 20-fold molar excess of DW0254). Conditions analyzed included P12-ICHIKAWA cells that were treated with PAL (1 µM) ± DW0254 (20 µM) prior UV irradiation, streptavidin pulldown and label-free differential quantitative mass spectrometry analysis. Three biological replicates for each sample were performed. bottom: Summary of the significant protein target hits of PAL identified in P12-ICHIKAWA; Proteins with an adjusted *p*-value (or *q* value) <5% and a FC of >2 were selected to be differentially modulated. A protein was considered as a hit of DW0254 when identified with at least two peptides in minimum two out of three replicates, FC > 2 and adjusted *p*-values < 0.05 in the two comparisons, PAL/DMSO and PAL/PAL + DW0254. **D** top: MS signal intensity of PDE6D in CCRF-CEM pulldown samples. Histogram plots represent quantitative determination of PDE6D MS signal intensity in the different pulldown samples (DMSO, PAL alone, and PAL in combination with a 20-fold molar excess of DW0254). *Y*-axis shows log2 value of protein identified. Proteins eluted from the beads were separated by SDS-PAGE and protein bands within the molecular weight range 15–18 kDa were excised. Proteins were prepared for downstream label-free differential quantitative mass spectrometry analysis. Three biological replicates for each sample were performed. bottom: Summary of the significant protein target hits of PAL identified in CCRF-CEM cells; Proteins with an adjusted p-value (or *q* value) <5% and a FC of >2 were selected to be differentially modulated. A protein was considered as a hit of DW0254 when identified with at least two peptides in minimum two out of three replicates, FC > 2 and adjusted *p*-values < 0.05 in the two comparisons, PAL/DMSO and PAL/PAL + DW0254. **E** In-gel fluorescence scanning showing the proteome reactivity profiles of live CCRF-CEM cells photolabeled by PAL (1 µM) with or without DW0254 (20 µM). FL: in-gel fluorescence scanning. CBB: Coomassie gel. **F** In-gel fluorescence scanning showing the recombinant human His-TEV-PDE6D-Avitag protein photolabeled by PAL (1 µM) with or without DW0254 (20 µM). FL: in-gel fluorescence scanning. CBB: Coomassie gel. **G** Single-stage LC-MS (MS1) intensity values of PAL-modified peptide TGKILWQGTED following His-TEV-PDE6D-Avitag protein labeling with PAL in competition with DW0254. The peptide adduct was identified in the sample irradiated with PAL in the presence of DW0254 but with a peak intensity >4-fold lower compared to the sample irradiated with PAL alone. **H** The second stage of mass spectrometry (MS2) for the PAL-modified peptide TGKILWQGTED of His-TEV-PDE6D-Avitag protein. MS2 spectra of the probe-modified peptide 1827.9216 *m/z* and its intact counterpart 1246.6193 *m/z*. Unlabeled fragment ions y1–y6 and b1–b9 were detected in both the PAL-modified peptide TGKILWQGTED and its intact counterpart. The fragment ion +178.12 *m/z* cleaved from PAL1 upon CID fragmentation was detected only in the MS2 spectrum of the PAL1-modified peptide.

Unexpectedly, and in contrast with NSC23766, neither DW0069 nor optimized DW0254 showed inhibition of the RAC1-TIAM1 protein-protein interaction as measured by homogeneous time-resolved fluorescence (HTRF) (Fig. 3A and Supplemental Fig. 1F). In comparison with the off-target effects exhibited by NSC23766, DW0069 chemical series showed no significant inhibition against a focused panel of kinases and G protein-coupled receptors (GPCRs) (Supplemental Fig. 1G) [21]. With certain key pathway targets ruled out, we embarked upon on the deconvolution of the putative molecular targets of DW0254 using cellular photoaffinity labeling methods combined with label-free quantitative mass spectrometry analysis (PAL-MS). A PAL photoprobe consisting of the DW0254 warhead covalently linked to a minimalist terminal propargyl-diazirine photocrosslinker [22] was synthesized (Fig. 3B). Like its parent compound, the PAL probe possessed antiproliferation properties (data not shown), demonstrating that the photoprobe was cell permeable and retained its activity.

Retinal rhodopsin-sensitive cGMP 3ʹ,5ʹ-cyclic phosphodiester 6 subunit delta (PDE6D) was identified as a target hit in P12-ICHIKAWA cells with the highest signal intensity (Log2 Intensity) of 24.66 and with the highest sequence coverage of 28.6% (Fig. 3C). One additional target, SEPT11 was identified with a lower signal intensity of 20.50 and sequence coverage of 8.1% (Fig. 3C). Due to the higher sequence coverage, we focused additional studies on PDE6D.

First, we confirmed PDE6D-PAL specific binding in an additional cell line, CCRF-CEM by band isolation with a high signal intensity of 21.44 (Fig. 3D). Labeled protein patterns showed a protein band of ~17 kDa photolabeled with PAL probe that was protected by excess DW0254 (Fig. 3E). Photolabeling of recombinant human PDE6D expressed in *E. coli* also confirmed photoincorporation of the PAL probe into PDE6D that was fully protected by an excess of DW0254 (Fig. 3F).

Next to gain insights into the binding site through identification of the specific photolabeled residues, recombinant PDE6D was UV-irradiated alone or with PAL probe in the presence or absence of DW0254 and analyzed by liquid chromatography–mass spectrometry (LC-MS/MS). A unique tryptic peptide of human PDE6D, TGKILWQGTED, was detected with an increase in peptide mass of +581.2997 *m/z* corresponding to the incorporation of the PAL probe, with a >4-fold lower peak intensity in the presence of the competitor DW0254 (Fig. 3G). PAL probe-modified peptide and its unlabeled control MS data were manually evaluated for the presence of specific probe-labeled b- or y-type fragment ions to further refine the localization of the photoadduct to a specific amino acid. Fragment ions y1-y6 and b1-b9 were detected in the unlabeled control TGKILWQGTED peptide (Fig. 3H, top). The PAL-modified peptide (Fig. 3H, bottom) shared the same fragment ions except for y1 and y2 suggesting that the PAL probe photolabeled, in a DW0254-inhibitable manner, residues E36 or D37 within the hydrophobic pocket of the molecule.

### Saturating mutagenesis screen hints at DW0254 binding mode

**Fig. 4:**
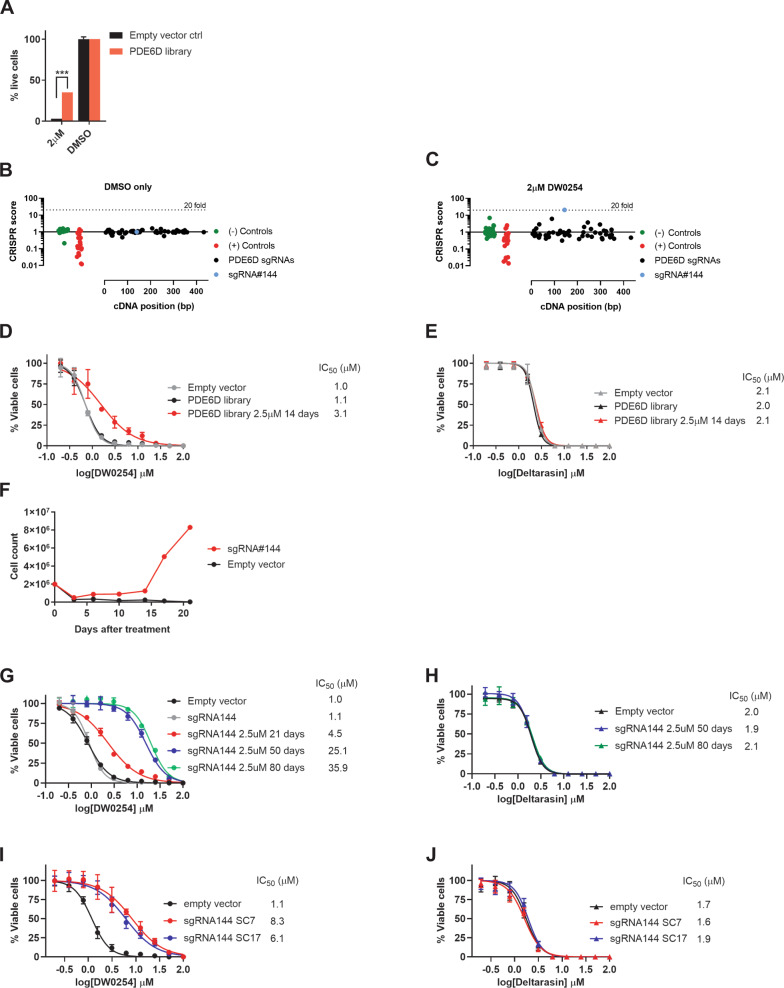
Identification of mutations on V49 and neighboring residues of PDE6D hydrophobic domain as essential for cellular resistance to DW0254. **A** Percentage of live P12-ICHIKAWA cells by DAPI staining after transduction with either PDE6D library or empty vector control treated for two weeks with 2 μM of DW0254 or DMSO, data represent mean ± SD of three technical replicates, ***P ≤ 0.001. **B** Changes in barcoded sgRNAs of untreated PDE6D library cells 14 days after transduction. The cDNA position (in bp) is shown on the *X*-axis. The fold-change in CRISPR score is shown on the *Y*-axis. Negative and positive controls are shown in green and red dots, respectively. Negative controls used were non targeting sgRNAs and positive controls targeting essential genes, including PCNA, CDK1, CDK9, RPA3, BRD4, MYC, and RPS20. **C** Changes in barcoded sgRNAs of PDE6D library cells treated for 14 days with 2 μM of DW0254 versus 14 days of DMSO. Dotted line on panels (**B**) and (**C**) represents a 20-fold change on CRISPR score. **D** DW0254 dose response curves showing % of viable PDE6D library cells or controls untreated or treated with DW0254 at 2 µM for 14 days. **E** Deltarasin dose response curves showing % of viable empty vector transduced cells, untreated PDE6D library cells and PDE6D library cells treated with DW0254 at 2 µM for 14 days. **F** Cell growth curves for P12-ICHIKAWA cells expressing Cas9 only or Cas9 and sgRNA144, treated with 2.5 μM of DW0254 for 21 days. **G** DW0254 dose response curves showing % of viable empty vector transduced cells, untreated sgRNA144 transduced cells, and sgRNA144 cells treated with DW0254 for 21, 50, and 80 days. **H** Deltarasin dose response curves showing % of viable untreated sgRNA144 transduced cells and controls, and sgRNA144 cells treated with DW0254 for 21, 50, and 80 days; For panels (**D**), (**E**), (**G**) and (**H**): data represent mean ± SD of two independent experiments with *N* = 3 samples for each condition. **I** DW0254 dose response curves showing % of viable cells transduced with empty vector and two single cell clones of sgRNA144 transduced cells. **J** Deltarasin dose response curves showing % of viable empty vector and two single cell clones of sgRNA144 transduced cells; For panels (**I**) and (**J**) data represent mean ± SD of two independent experiments with *N* = 4 samples for each condition.

To further validate PDE6D as the biological target, to identify additional key residues for binding, and to link target engagement to the observed phenotype, we designed a sgRNA library (Supplemental Table 1) and performed a saturation mutagenesis screen of PDE6D. spCas9-expressing P12-ICHIKAWA cells were transduced with the PDE6D sgRNA library and treated with DW0254 with the goal of selecting resistant cells. After 2 weeks of treatment, 35% of library-transduced cells were alive, compared to 3% of the empty vector control cells (Fig. 4A). A robust editing efficiency was confirmed by the decrease in positive control sgRNAs that targeted essential genes (Fig. 4B). Specific sgRNAs were significantly enriched after DW0254 treatment, including sgRNA#144, which was identified at 20-fold increased frequency relative to DMSO-treated cells (Fig. 4C, blue dot) and cells exhibited a ~3-fold higher IC_50_ when compared to untreated library transduced cells (Fig. 4D), confirming decreased compound sensitivity. Deltrasin, a commercially available PDE6D inhibitor, and additional derivative compounds have previously been reported to bind in PDE6D’s hydrophobic pocket and inhibit the growth of pancreatic cancer cell lines [23]. Since our data suggests the same binding site for DW0254, we tested DW0254-treated PDE6D-edited cells for sensitivity to Deltarasin. While unedited cells displayed a higher IC_50_ to Deltarasin (gray in Fig. 4E) when compared to DW0254 (gray in Fig. 4D), DW0254-treated PDE6D-edited cells demonstrated no increased resistance to Deltarasin (Fig. 4E).

Next, to define the mutations generated with sgRNA#144 and confirm their association with resistance to DW0254, we transduced spCas9 expressing cells with sgRNA#144 alone. Resulting edited cells demonstrated a resistance phenotype as early as 10 days after treatment, with a fivefold increase in cell counts when compared to controls, and robust cell growth after day 14 (Fig. 4F). Importantly, 100% of empty vector control cells were dead after 17 days of treatment with DW0254 and no resistance was observed in this condition (Fig. 4F). Continued selection led to >30-fold increased IC_50_ (Fig. 4G). Although highly resistant to DW0254, these edited cells showed no increased resistance to Deltarasin (Fig. 4H). Resistant cells genome was enriched for INDELS that would cause the deletion of V49 and neighboring residues within the hydrophobic pocket of PDE6D (data not shown). We validated these predicted mutations using long-range RT-PCR and documented a −6 bp in-frame mutation that would cause the combined deletion of R48 and V49 residues (Supplemental Fig. 2B) and two out-of-frame mutations (+1 bp and −8 bp) that both lead to a frame-shift with the formation of a new open reading frame (ORF) with 124 and 127 instead of 150 residues, respectively (Supplemental Fig. 2C). Since the new ORF is predicted to translate into a protein that is missing a substantial portion of the hydrophobic pocket and such a change would most likely prevent correct protein folding, we focused our subsequent studies on R48del V49del PDE6D.

To definitively confirm the causal relationship of this mutation to the observed resistance to DW0254, we next isolated sgRNA#144 transduced single cell clones before treatment with DW0254. Edited single cell clones (SC7 and SC17) which harbored R48 and V49 deletions showed a 6–8-fold increased IC_50_ to DW0254 when compared to controls (Fig. 4I) while again showing no resistance to Deltarasin (Fig. 4J).

### Distinct binding of DW0254 to PDE6D hydrophobic pocket

**Fig. 5:**
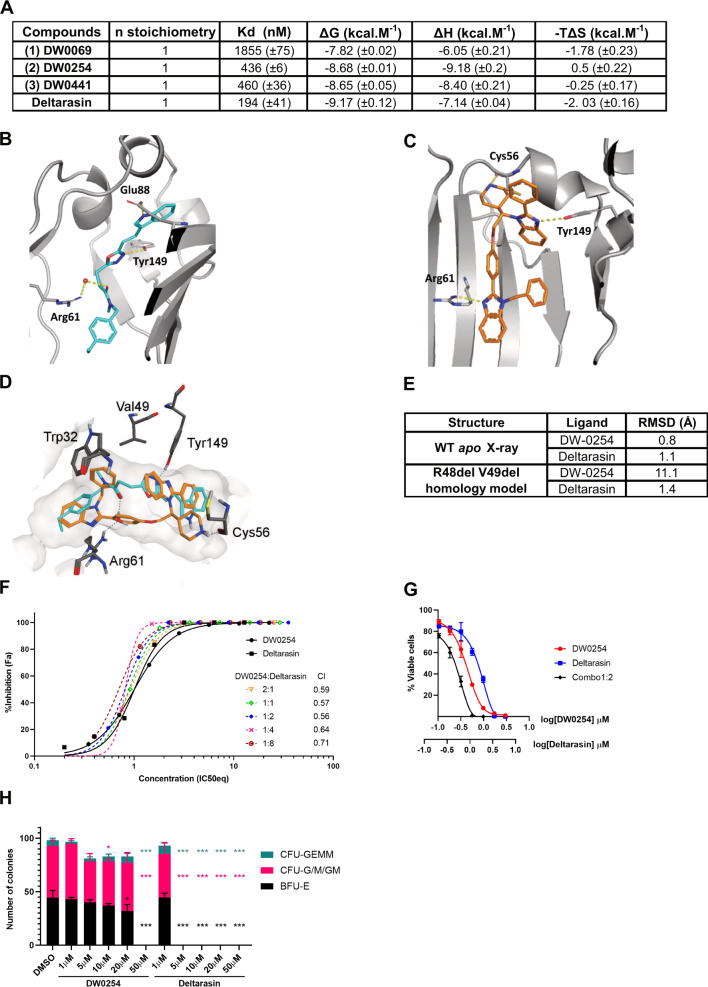
Co-crystallization of PDE6D and DW0254 confirms compound binding to hydrophobic pocket and evidences different binding modes between this inhibitor and Deltarasin. **A** Binding affinity (Kd) and thermodynamics parameters for ligand binding to PDE6D determined by Isothermal Titration Calorimetry (ITC). **B** The crystal structure of compound DW0254 in the PDE6D-binding pocket; Q88, R61, and Y149 are shown in sticks to highlight the hydrogen bind interactions**. C** The crystal structure of Deltarasin in the PDE6D-binding pocket; C56, R61, and Y149 are shown in sticks to highlight the hydrogen bind interactions. **D** Experimental binding mode of DW-0254 (cyan) in wild type PDE6D. The superposed 3D coordinates of Deltarasin (orange) are also shown. Several binding site residues, including V49, are shown for reference. **E** Docking results in wild type apo structure of PDE6D and R48delV49del mutant; Root mean square deviation (RMSD) with respect to 3D coordinates of the ligands in the superposed X-ray complex of the wild type protein are reported. **F** Drug dosage curves for P12-ICHIKAWA cells treated with DW0254 alone, Deltarasin alone, or various combinations of both obtained from a full matrix using the “Fixed Ratio” Method; data show mean ± SD of *n* = 3 samples for each condition, and combination indexes for each combo calculated using the Chou-Talalay theorem. **G** Drug dosage curves for P12-ICHIKAWA cells treated with DW0254 alone, Deltarasin alone, or the combination of both at a 1:2 ratio; data show mean ± SD of *n* = 3 samples for each condition. **H** Colony counts of healthy human CD34^+^ cells after 14 days culture in MethoCult H4435 enriched medium in presence of DMSO or increasing concentrations of DW0254 or Deltarasin. CFU-GEMM: Colony-forming unit—granulocyte, erythroid, macrophage, megakaryocyte; CFU-GM: Colony-forming unit—granulocyte, macrophage; BFUE: Burst-forming unit—erythroid. Data represent mean ± SD, *n* = 3 samples for each condition. *p < 0.05; ***p < 0.001.

Next, we determined the binding affinities of the various compounds by isothermal calorimetry (ITC) using recombinant PDE6D protein. For the DW compounds, ITC binding affinity is in line with the order of cellular activities while Deltarasin showed a slightly higher affinity to the protein when compared to DW0254 (Fig. 5A). Inactive DW0346 showed very weak affinity with Kd 68.5 µM by ITC. Cocrystal structure of DW0254 with recombinant PDE6D shows the small molecule bound inside the hydrophobic pocket, with hydrogen bond interactions via glutamine Q88, tyrosine Y149 and arginine R61, the latter interaction being water mediated (Fig. 5B). Deltarasin can occupy the same pocket undergoing hydrogen bonding with the same residues R61 and Y149, but also with cysteine C56 (Fig. 5C), which differentiates it from the interactions observed for DW0254. The observed network of hydrogen bonding with the protein backbone supports the strong enthalpy (ΔH) driven binding for both molecules as observed by ITC (Fig. 5A).

Guided by the crystallographic information we were also able to postulate a binding pose for the PAL probe (Supplemental Fig. 3). To contextualise the crystallographic binding modes with the saturating mutagenesis screen results, superimposing the binding poses of DW0254 and Deltarasin highlighted that V49 defines the shape of the pocket (light gray area, Fig. 5D), and establishes hydrophobic contacts only with DW0254 (cyan) but not Deltarasin (orange). In addition, in silico docking of DW0254 to R48del V49del PDE6D confirmed an accentuated increase in the root mean square deviation (RMSD) in contrast with Deltarasin’s RMSD that was only marginally affected (Fig. 5E), strongly suggesting DW0254 would be unlikely to bind PDE6D in the event of deletion of these two residues. Interestingly, the combination of DW0254 and Deltarasin had a synergistic effect in vitro (Fig. 5F) with the lowest combination index at a 1:2 range (Fig. 5G), suggesting that even though binding of these compounds to PDE6D is mutually exclusive, they may target different protein conformations more efficiently. However, while DW0254 exhibited low toxicity to CD34^+^ healthy donor cells at therapeutic dosages, Deltarasin showed decreased colony counts even at low dosages (Fig. 5H) indicating possible off-target effects of the latter.

### RAS protein dynamics and downstream effects of DW0254

**Fig. 6:**
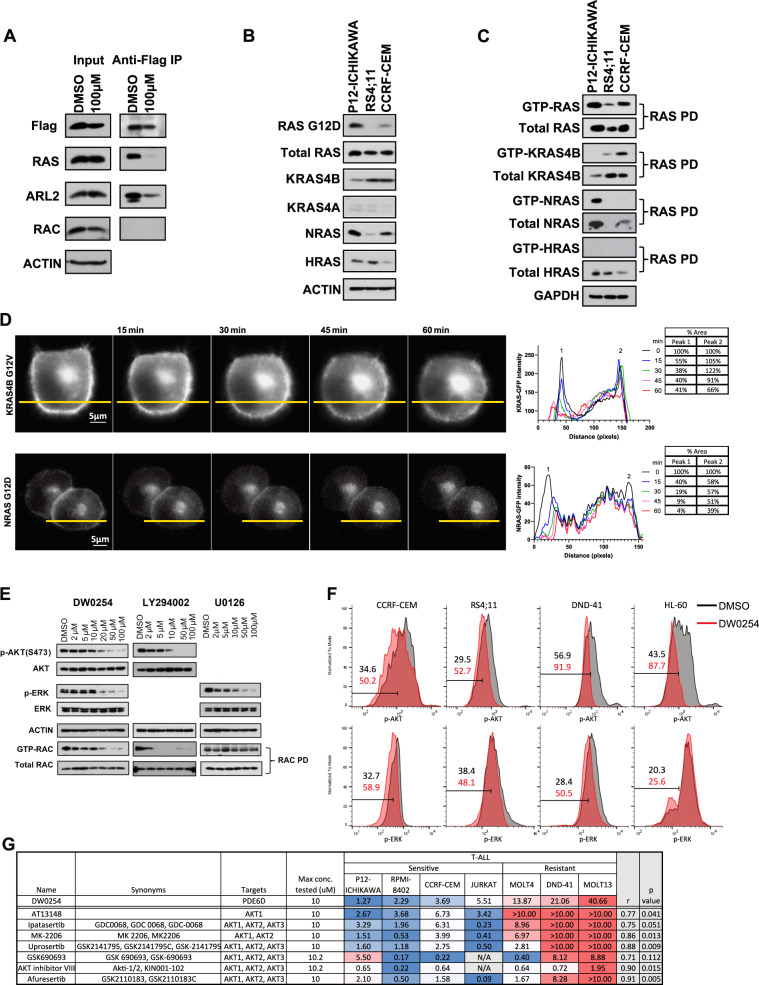
The expression and activation of RAS isoforms in DW0254 sensitive leukemia cells and the effects of DW0254 on PDE6D/RAS interaction and RAS subcellular location. **A** DW0254 treatment inhibits the binding of PDE6D to RAS and ARL2 in P12-ICHIKAWA cells. Co-immunoprecipitation (CoIP) was performed with an anti-FLAG antibody (F1804, Sigma-Aldrich) on lysates from FLAG-tagged PDE6D transduced P12-ICHIKAWA cells treated with 100 µM DW0254 or DMSO. Cell lysates (Input) and protein eluted from beads (IP) were analyzed by Western blotting with anti-Flag, anti-RAS (05-1072, Millipore Sigma, Billerica, MA), anti-ARL2 (ab183510, Abcam, Cambridge, MA), anti-RAC (610651, BD) antibodies. **B** The expression pattern of RAS isoforms in leukemia cell lines sensitive to DW0254. Western blot analysis of whole-cell lysates from P12-ICHIKAWA, RS4;11, and CCRF-CEM leukemia cells detected by anti-RASG12D (14429S, Cell signaling), anti-RAS (05-1072, Millipore Sigma), Anti-KRAS4B (WH0003845M1, Millipore Sigma), anti-KRAS4A (ABC1442, Millipore Sigma), anti-NRAS (sc-31, Santa Cruz), and anti-HRAS (18295-1-AP, Proteintech, Rosemont, IL) antibodies. **C** Activated RAS isoforms in P12-ICHIKAWA, CCRF-CEM and RS4;11 cells. GST pulldown assays were performed by incubating protein lysates prepared from P12-ICHIKAWA, CCRF-CEM and RS4;11 with RAF-1 RBD conjugated agarose beads. The GTP-RAS proteins bound to the beads or the whole cell lysates to detect the level of total RAS protein were identified using anti-RAS, anti-KRAS4B, anti-NRAS, or anti-HRAS antibodies described above. For **A**, **B**, and **C**, beta-ACTIN or GAPDH (A300-641A, BETHYL, Montgomery, TX) were used as a protein loading control, one representative experiment of two or three is shown. **D** Mislocalization from cell surface membrane of GFP-tagged mutant KRAS4BG12V (upper panel) or NRASG12D (lower panel) in PANC-1 cells after treatment with 20 µM of DW0254. Time in minutes is indicated above the panels; the first panel represents the moment immediately after the addition of the inhibitor. The intensity profiles on the right show changes in fluorescence along the *X* axis, represented by yellow lines on the micrographs. Tables show changes in percentage of the area under the peaks, where time 0 peaks represent the intersection of the *X* axis with the cell membrane. **E** Western blot showing total and phosphorylated AKT and ERK, and pulldown results for RAC activation in P12-ICHIKAWA cells treated with increasing doses of DW0254, PI3K inhibitor LY294002, or MEK inhibitor U0126 for 3 h. Total AKT, and ERK expression were assessed using anti-AKT (9272, Cell signaling) and anti-ERK (9102S, Cell signaling) antibody respectively. Phosphorylation of AKT and ERK were assessed using anti-phospho-AKT Ser473 (9271S, Cell signaling) and anti-phospho-ERK (4377S, Cell signaling) antibody respectively. Total RAC and GTP-RAC were analyzed by RAC pull-down assay as described in Fig. 2B. For **A**, **B**, **C**, and **D**, beta-ACTIN or GAPDH were used as a protein loading control. Data represent three independent experiments. **F** PhosphoFlow results showing intensity of p-AKT (top) and p-ERK (bottom) intracellular staining on cells treated with DMSO only (black) or 50 µM DW0254 (red) for 1 h. Data are representative of two independent experiments with four cell lines showing the same results. **G** Summary of response to AKT inhibitors in T-ALL cell lines from “The Genomics of Drug Sensitivity in Cancer Project” database compared to sensitivity to DW0254. Pearson’s correlation coefficient (*r*) and the *p* value for the correlation coefficient between response to DW0254 and each AKT inhibitor are shown on the right extremity.

PDE6D has been shown to bind farnesylated RAS proteins and facilitate their trafficking and plasma membrane (PM) localization [24, 25]. To determine the effect of DW0254 on PDE6D-RAS interactions, we generated P12-ICHIKAWA cells that stably expressed a FLAG-tagged human PDE6D protein. Co-immunoprecipitation studies confirmed PDE6D binding to both RAS and ADP-ribosylation factor-like protein 2 (ARL2) protein essential for cargo displacement, that decreased after treatment with DW0254 (Fig. 6A). No direct binding was observed between PDE6D and RAC (Fig. 6A).

PDE6D has been reported to chaperone NRAS, HRAS, and KRAS4B but not KRAS4A [24, 25]. While all RAS isoforms were detected in a panel of DW0254-sensitive ALL cell lines, the most abundantly activated isoforms were NRAS in P12-ICHIKAWA and KRAS4B in RS4;11 and CCRF-CEM (Fig. 6B, C). To further test whether the DW0254-dependent disruption of the interaction between PDE6D and RAS is associated with altered subcellular localization of RAS, we used fluorescently tagged mutant RAS proteins to analyze RAS localization before and after treatment with DW0254. Transfection of RAS proteins into suspension leukemia cells has proven to be very difficult and often leads to apoptosis and loss of adherence. To address this, PANC-1 cell lines were chosen for live imaging studies for their adherence characteristics and because they were previously used by others to show delocalization of RAS from the cell membrane upon PDE6D inhibition [23]. Live-cell fluorescence imaging demonstrated that mutant KRAS4B and NRAS dissociated from the PM after DW0254 treatment (Fig. 6D).

Biological effects of RAS proteins are exerted from the PM through the activation of kinase pathways including PI3K/AKT and MAPK/ERK, which are commonly constitutively activated in cancer [26]. We observed decreased activation of PI3K/AKT and MAPK/ERK pathways, measured by levels of phospho-AKT and phospho-ERK, upon inhibition of PDE6D-RAS interaction by DW0254 (Fig. 6E, left panel). Next, to establish a biochemical link between RAC and RAS pathway inhibition we examined if RAC activation is affected by the inhibition of PI3K/AKT or MEK/ERK pathways. The PI3K inhibitor LY294002 clearly decreased GTP-RAC levels while the MEK inhibitor U0126 had no demonstrable effect on RAC activity (Fig. 6E). We further analyzed the levels of phospho-AKT and phospho-ERK in a panel of sensitive and resistant cell lines and observed a consistent decrease in phosphorylation levels of both proteins in all the cell lines tested (Fig. 6F). Taken together these results provide a potential molecular link between PDE6D pocket occupation by DW0254, RAS mislocalization, decreased downstream pathway activation, and inhibition of RAC activity.

Next, we used “The Genomics of Drug Sensitivity in Cancer Project” platform [27] to study a possible correlation between the response to kinase inhibitors and sensitivity to DW0254. We observed a positive correlation between the response to most AKT inhibitors and DW0254 in T-ALL cell lines (Fig. 6G). The same was not true for ERK inhibitors (data not shown). There was insufficient data on myeloid lines to carry out an analogous comparison.

### DW0254 anti-leukemic activity in a murine xenograft model

**Fig. 7:**
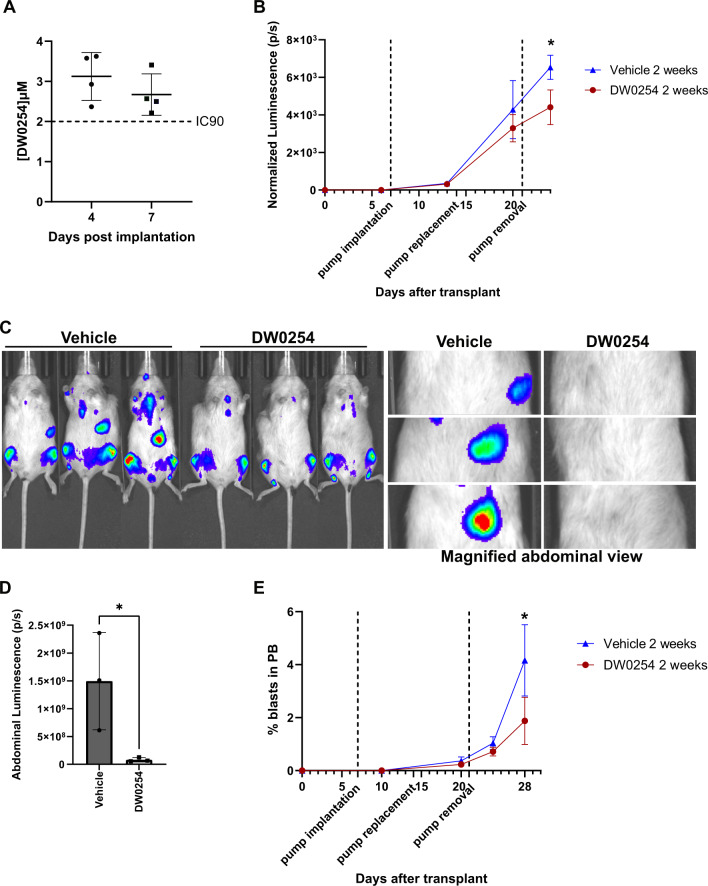
In vivo treatment of mice with DW0254 using Alzet osmotic pumps. **A** Plasma concentration of DW0254 (µM) at day 4 and 7 post subcutaneous implantation of Alzet osmotic pumps 2001 containing a 500 mg/ml solution of DW0254 in water with 50% DMSO and 15% Ethanol with a 7-days pumping rate of 1 µl/h. Individual values as well as mean ± SD of four animals are shown. **B** Changes in luminescent signal in units of photons/s (p/s), after luciferase expressing P12-ICHIKAWA xenograft for mice implanted with DW0254 or vehicle control pumps at day 7 and replaced at day 14 post-transplant. Photon flux signal after pump implantation was normalized over signal before implantation for each mouse. **C** Bioluminescent images of day 24 post-transplant (last data point of panel **B**) of mice treated with DW0254 or Vehicle control pumps from day 7 to day 21 and magnified abdominal view showing increased spleen signal in control mice. **D** Bar graph showing quantification of abdominal bioluminescence depicted in panel (**C**) right. Welch’s correction *t*-test was used for statistical analysis since variances between the groups were significantly different. **E** Percentage of blasts in the peripheral blood (PB) of mice treated with DW0254 or Vehicle control pumps for 2 weeks. Data in **B**, **D**, and **E** represent mean ± SD, *n* = 3 animals for each condition. *p < 0.05.

Initial in vivo pharmacokinetics (PKs) assays demonstrated low solubility and rapid plasma clearance of DW0254 (data not shown) which meant direct in vivo administration by IP or gavage could not be performed. First, we examined the antitumor effects of PDE6D inhibition on leukemia cell growth ex vivo by treating luciferase-expressing P12-ICHIKAWA cells with DW0254 before transplantation into sub-lethally irradiated non-obese diabetic severe combined immunodeficiency-gamma (NSG) mice. Disease burden as assessed using bioluminescence imaging was significantly reduced in mice injected with DW0254 treated cells compared to the vehicle control group on days 13, 16, 21, and 24 after injection (Supplemental Fig. 4). Next, we exploited a drug delivery strategy using Alzet osmotic pumps. Pumps with a 500 mg/ml solution of DW0254 were subcutaneously implanted in NBSGW mice. The concentration of DW0254 in the plasma was found to be higher than 2 µM (IC_90_ for P12-ICHIKAWA cells) over the course of the 7-days treatment (Fig. 7A). Subsequently, we used a luciferase-expressing P12-ICHIKAWA xenograft model to test drug efficiency in vivo. Pumps with 500 mg/ml DW0254 or vehicle only were implanted in mice at day 7 post-transplant and replaced at day 14 to allow for a 2-week cumulative treatment. Mice treated with DW0254 showed decreased tumor progression when compared to controls (Fig. 7B). Notably, at day 24 control mice exhibited luminescent signal in the abdominal area corresponding to the spleen while DW0254-treated mice demonstrated no observable signal (Fig. 7C, D). Blasts were found in the peripheral blood of all mice from day 20 onwards with DW0254-treated mice showing decreased blast counts when compared to controls—1.8% compared to 4.2% on day 28 (Fig. 7E).

## Discussion

The results presented here provide evidence of the importance of PDE6D in sustaining downstream RAS signaling and cell survival in a large panel of acute leukemia cell lines.

Treatment of acute leukemia cell lines with DW0254 resulted in a clear decrease in GTP RAC. However, binding between DW0254 and RAC was not observed contradicting computer-aided drug design methodologies. We determined the direct target of DW0254 to be PDE6D, a chaperone protein that facilitates cytoplasmic trafficking of farnesylated molecules, including RAS, as a target for this compound, thus linking RAS transport with RAC GTPase activation in leukemic cells. Saturating mutagenesis experiments showed that the deletion of R48 and V49 residues causes changes to PDE6D pocket that prevent binding to DW0254 and result in resistance to the compound. The binding mode for DW0254 in PDE6D farnesyl binding pocket was also confirmed by crystallography and is different than the binding mode of another previously described inhibitor, Deltarasin. Further emphasizing the importance of this difference, R48del V49del edited cells are not resistant to Deltarasin. Interestingly, the combination of DW0254 and Deltarasin had a significant synergistic effect suggesting that these compounds might be targeting singular conformations of PDE6D with different efficiencies. Indeed, large conformational changes in PDE6D to facilitate the binding of farnesylated RAS proteins deeper within the hydrophobic pocket have been previously described [28]. In addition, DW0254 did not show any toxicity to CD34^+^ healthy donor cells at therapeutic levels, suggesting a potential for translational improvement of this inhibitor. Even though a role for PDE6D on blood cell differentiation has not been previously described, low dosages of Deltarasin led to decreased colony counts.

DW0254 treatment leads to the delocalization of RAS from the plasma membrane, where it can activate downstream factors [29], to the cytoplasm, as had been previously reported with other PDE6D inhibitors [23]. As shown here and in line with recent studies on the importance of RAS membrane localization [29], RAS delocalization ultimately results in an inability to activate target pathways including MAPK/ERK, PI3K/AKT, and consequently RAC. However, we observed the same range of inhibition of RAS downstream pathways upon DW0254 treatment in both sensitive and resistant cell lines. This result implies that kinase pathways inhibition downstream of RAS delocalization does not uniformly associate with decreased cell viability in acute leukemia cell lines. Nonetheless, sensitivity to DW0254 positively correlates with response to AKT inhibitors, at least in T-ALL cell lines, suggesting that PDE6D inhibition effectively triggers a downstream anti-leukemic response in AKT dependent cells. Together with the fact that DW0254 compound sensitivity did not correlate with RAS mutational status alone of acute leukemia cell lines, this suggests that RAS/AKT/RAC pathway activation might be a better predictor of response to PDE6D inhibitors. Regardless, since PDE6D acts as a carrier for additional prenylated cargo, we cannot exclude that its hydrophobic pocket occupancy by DW0254 might affect additional RAS-independent mechanisms of PI3K/AKT/RAC activation [30–32].

Importantly, we were able to recapitulate the antileukemic results observed in vitro, in a leukemia xenograft model showing decreased tumor progression in DW0254 treated mice, with decreased tumor burden in the spleen and decreased blast counts in the periphery. However, residual levels of precipitated drug were observed after pump removal in some mice (data not shown) highlighting the need for further medicinal chemistry efforts to improve the aqueous solubility of the compound.

In conclusion, we have validated the RAS chaperone PDE6D as a novel molecular target for aggressive leukemias. We have derived a series of compounds with demonstrated PDE6D inhibition that bind to its hydrophobic pocket differently from a previously identified inhibitor series showing little toxicity to normal human and mouse hematopoietic progenitor cells. The binding of DW0254 to PDE6D resulted in delocalization of RAS from the membrane and consequent inhibition of major pro-survival pathways including MAPK/ERK, PI3K/AKT and downstream RAC activation. Whether or not targeting RAC is an effective therapeutic approach in RAS mutant and/or AKT dependent leukemias needs further analysis with RAC-specific inhibitors. From a clinical standpoint, the fact that PDE6D inhibition efficiently triggered an antileukemic response in AKT dependent cell lines independently of their RAS mutational status, emphasizes the importance of studying pathway dependency in cancer cells rather than focusing on their mutational landscape when deciding which inhibitors to use.

## Methods

### Cell lines

CCRF-CEM, RS4;11, MV4;11, and PANC-1 cells were obtained from ATCC and all others from DSMZ. Cells were cultured according to suppliers’ instructions and periodically tested for the presence of mycoplasma.

### Cell viability assay

Cells were treated for 3 days at 1 × 10^5^ cells/ml with limiting dilutions of DW0254 or DMSO only. On day 3, cells were stained with DAPI at a 1 μg/ml final concentration and the number of viable (DAPI-) cells in 25 μl of media were counted using BD LSR II.

### AnnV/PI staining and cell cycle analysis

P12-ICHIKAWA cells were plated at a 2 × 10^5^ cells/ml concentration with DW0254 or DMSO for 3 days. Cells were labeled with Dead Cell Apoptosis Kit with Annexin V FITC and PI (Thermo Fisher) or fixed in 70% ethanol at 4 °C overnight, followed by incubation with 10 µg/mL Ribonuclease A (Sigma-Aldrich, St Louis, MO) and 50 µg/mL PI (BD Biosciences PharMingen, San Diego, USA) or 10 µg/mL DAPI (Thermo Fisher). Flow cytometry analysis was performed on a BD LSR II.

### Generational cell tracing

Cells were stained with CellTrace™ Far Red (Thermo Fisher) following manufacturer’s instructions and incubated with DW0254 or DMSO. Cells were analyzed on BD LSR II after 15 min (Time 0) and the following 3 days at the same time.

### Recombinant protein expression and purification

Recombinant human Rac1 (Q2-L177) with TEV-protease cleavable 6xHis-tag fused to its N-terminus, and truncated recombinant human Tiam1 (R1033-E1406) with FLAG-tag fused to its N-terminus were cloned into in the pTriIJ-HV vector and expressed in BL21 (DE3). Rac1 protein went through a nickel affinity column followed by a Resource Q column and finally Superdex 75 (GE Healthcare) before concentration to 25 mg/ml. Tiam1 protein was purified using the ANTI-FLAG® M2 affinity gel (Sigma-Aldrich) followed by Superdex 75. Recombinant human PDE6D (S2-V150) with TEV-protease cleavable 6xHis-tag fused to its N-terminus, was cloned into pET28a, expressed in BL21-CodonPlus (DE3)-RIL and purified using nickel affinity chromatography followed by TEV protease cleavage, tag removal, and finally Superdex 75 before concentration to 13 mg/ml.

### Isothermal calorimetry (ITC)

PDE6D was dialyzed in buffer (20 mM HEPES pH7.3, 150 mM NaCl, 1 mM TCEP) at 4 °C, overnight. Titrations were carried out on an iTC200 calorimeter (MicroCal Inc). PDE6D (200 µM with 2% DMSO) was titrated into small molecule in the cell (20 µM in degassed dialysis buffer with 2% DMSO final) and data were analyzed using Origin (OriginLab Corp.) and fitted by using a single-site binding model.

### Rac1-Tiam1 homogeneous time-resolved fluorescence assay (HTRF)

30 nM His-tagged Rac1 protein was pre-incubated with compound at room temperature in assay buffer (50 mM Hepes (pH 7.6), 100 mM NaCl, 1 mM DTT, 10 mM MgCl_2_, 0.1% Nonidet P-40). After 30 min pre-incubation, 300 nM FLAG-tagged Tiam1, 2 nM anti-His-Eu3+, 20 nM anti-FLAG-XL665 were added. After 60 minutes RT incubation, 500 mM potassium fluoride (KF) was added and the reaction was measured after 30 minutes with EnVision 2104 Multilabel Reader (Perkin Elmer) with the following settings. Ex: 320 nm; Em1: 615 nm; Em2: 665 nm; Dichroic Mirror: D400.

### High density sgRNA library of human PDE6D

sgRNA sequences targeting the coding regions of human PDE6D (NM_002601.3) were designed using Genetic Perturbation Platform from Broad Institute [33] (Supplemental Table 1). Briefly, sgRNA oligonucleotides were synthesized via microarray (CustomArray) and cloned into the ipUSEPR lentiviral sgRNA vector that co-expresses a puromycin-resistant gene [puro^R^] and a red fluorescent protein [tagRFP]. The PDE6D scan library contains 116 unique sgRNA was packaged by HEK293 cells (ATCC) co-transfected with psPAX2 (Addgene) and pMD2.G (Addgene) to produce lentiviral particles. The lentiviral library was pre-titrated to obtain 5–10% infection (monitored by flow cytometry for tagRFP expression from ipUSEPR) in P12-ICHIKAWA spCas9 expressing cells. Each screen culture was calculated to maintain at least 1000× of the number of constructs in the library. The infected cultures were selected by sorting of RFP^+^ cells 3 days after transduction and expanded in supplemented media with puromycin (2.5 µg/ml; InvivoGen) and blasticidin (1 µg/ml; InvivoGen) for 3 additional days. Finally, selected cells were pelleted (day 0) and cultured in DMSO or 2.0 µM DW0245. After 14 days treatment cells were again pelleted. For sequencing sgRNAs, the genomic DNA of the screened cell pellets was harvested, PCR-amplified (NEBNext Ultra II Q5; NEB) using primers DCF01 5ʹ-CTTGTGGAAAGGACGAAACACCG-3ʹ and DCR03 5ʹ-CCTAGGAACAGCGGTTTAAAAAAGC-3ʹ and subjected to single-end 75 bp (SE75) high-throughput sequencing using a NextSeq550 (Illumina).

To quantify sgRNA reads in the library, we first extracted 20-nucleotide sequences that matched the sgRNA backbone structure (5ʹ prime CACCG and 3ʹ prime GTTT) from raw fastq reads. Extracted reads were then mapped to the PDE6D sgRNA library sequences using Bowtie2 [34]. Reads that were a perfect match to the reference were counted. The frequency for individual sgRNAs was calculated by the read counts of each sgRNA divided by the total read counts matched to the library. The CRISPR score was defined by the fold change of the frequency of individual sgRNAs between early (day 0) and late (defined time points) of the screened samples.

### Crystallization and structural determination

Native PDE6D crystals were grown by vapor diffusion at 22 °C by mixing equal volumes of protein with precipitant (0.1 M HEPES pH6.8–7.5, 20 mM MgCl_2_, 20 mM NiCl_2_ and 15–20% PEG3350). DW0254 and Deltarasin were incubated with PDE6D at 4 °C, at 4 mM and 1 mM final respectively. The PDE6D::Deltarasin complex was further concentrated to 19 mg/ml prior setting up the crystallization trays. PDE6D::Deltarasin and PDE6D::DW0254 complexes were grown by vapor diffusion at 22 °C in (0.1 M Sodium acetate pH4.0–4.5 and 28−30% PEG3350) and (0.1 M HEPES pH6.8–7.5, 20 mM MgCl_2_, 20 mM NiCl_2_ and 15–20% PEG3350) respectively. Prior to freezing in liquid nitrogen, crystals were cryoprotected by brief transfer to a solution of crystallization condition reservoir supplemented with 25% glycerol. Data were collected on beamlines at Diamond Light Source (Oxford, U.K.) and ALBA (Barcelona, Spain). Data were processed using XDS, xia or DIALS. Molecular replacement was performed using PHASER (using PDB code 5NAL as a reference model), and the refinement was performed with refmac5, buster and COOT. Compound dictionaries were generated using AFITT.

### Combination index analysis

Each drug was used alone or in combination at a concentration approximately equal to its IC50 and at concentrations within 2–2.5-fold increments above or below. Each data point was performed in triplicates. In this model, combination index (CI) scores estimate the interaction between the two drugs. If CI < 1, the drugs have a synergistic effect [35]. To allow a direct comparison of the dose-response curves, each drug concentration was normalized to its own IC_50_ value and named IC_50_ equivalent (IC50eq) as previously described by Zhao et al. [36]:

$${{{\mathrm{IC}}}}50{{{\mathrm{eq}}}} = \frac{{{{{\mathrm{C}}}}_{{{{\mathrm{a}}}},{{{\mathrm{x}}}}}}}{{{{{\mathrm{IC}}}}_{50,{{{\mathrm{a}}}}}}} + \frac{{{{{\mathrm{C}}}}_{{{{\mathrm{b}}}},{{{\mathrm{x}}}}}}}{{{{{\mathrm{IC}}}}_{50,{{{\mathrm{b}}}}}}}.$$

### PDE6D co-immunoprecipitation (Co-IP)

NH3-terminal FLAG-tagged human PDE6D was constructed by PCR, checked by sequencing, and subcloned into the BglII and EcoRI site of MSCV-IRES-GFP vector. GFP^+^ P12-ICHIKAWA cells were sorted 48 hours after lentiviral infection.

Cells with stable expression of FLAG-tagged human PDE6D were lysed in 1X cell lysis buffer (#9803, Cell Signaling, Danvers, MA) and incubated with anti-FLAG M2 Affinity Gel (A2220, Sigma-Aldrich) overnight at 4 °C. Protein complexes were washed five times with 1 mL lysis buffer, then 2X SDS sample buffer was added, following 100 °C incubation for 5 min.

### RAS and RAC activity assay

RAS and RAC activity were measured using a RAF-1 RBD and PAK-1 PDB pull-down assay kits respectively (Cat#17218 and Cat#14325, Millipore Sigma) following manufacturer’s instructions. For comparison with total corresponding protein, 5–10% of total lysates used for pulldown were loaded to adjacent wells.

### Transfection and fluorescent imaging

PANC-1 cells were collected from a confluent flask, split 1:5 and plated on 35 mM µ-Dishes with a polymer coverslip bottom (Ibidi) and incubated in a humidified 37 °C incubator with 5% CO_2_ for 24 h. The next day cells were transfected with pEGFP-C3 KRAS4B 12 V or pEGFP-C3 NRAS 12D using Lipofectamine 3000 (Thermo Fisher) following manufacturer’s instructions, and incubated for 3 days in a humidified 37 °C incubator with 5% CO_2_. Cells in 1.8 ml PBS with 10%FCS were imaged in a Nikon Eclipse Ti inverted microscope with a humidified live cell imaging chamber using NIS-Elements software. 200 µl of PBS with 10% DMSO only or of 200 µM DW0254 previously diluted in PBS with 10% DMSO were added, and samples were imaged every 5 min for 1 h.

### PhosphoFlow

Cells were incubated for 1 h with 50 µM DW0254 or DMSO only in complete media, fixed immediately with BD Cytofix Buffer and permiabilized with BD Perm buffer III according to suppliers’ instructions. After wash, 1 × 10^6^ cells were resuspended in 100 µl and stained with 4 µl BD Phosflow™ PE Mouse Anti-ERK1/2 (pT202/pY204) or anti-Akt (pS473) and analyzed using BD LSR II.

### Alzet osmotic pump implantation and bioluminescent imaging for DW0254 in vivo efficacy studies

To generate a cell line with luciferase expression, P12-ICHIKAWA cells were infected with Lenti-FUW-Luc-mCh-puro virus and selected in liquid culture with puromycin (Sigma-Aldrich) 2.5 µg/mL for 7 days following mCherry^+^ cell sorting.

All animal studies were approved by the Boston Children’s Hospital or Dana-Farber Cancer Institute Animal Care and Use Committee. 6- to 8-week-old NOD.Cg-KitW-41J Tyr + Prkdcscid Il2rgtm1Wjl/ThomJ (NBSGW) mice (Jackson laboratories, Bar Harbor, ME) were injected with 5 × 10^5^ luciferase expressing P12-ICHIKAWA cells treated. On day 6 post-transplant mice were randomized by weight and bioluminescent signal using Randomice [37]. Alzet osmotic pumps 2001 (1 µl per hour, 7 days) containing 500 mg/ml DW0254 or vehicle (50% DMSO, 15% Ethanol in water) were subcutaneously implanted in mice. Disease burden was assessed using bioluminescence imaging every 4–8 days after injections. Prior to imaging, each mouse was given an intra-peritoneal (i.p.) injection of luciferin (PerkinElmer, Part Number #122799) at a dose of 150 mg/kg body weight. General anesthesia was then induced with 2.5% isoflurane and mice were placed in a supine position in the light-tight heated chamber; anesthesia was continued during the procedure with 2% isoflurane introduced via nose cone.

Optical images were displayed and analyzed with IVIS Living Image (Xenogen) software and optical signal was expressed as photon flux, in units of photons/s.

### PAL probe synthesis, photoaffinity labelling and LC-MS/MS

All the information regarding the synthesis of PAL probe, and specifics on photoaffinity labelling and LC-MS/MS data collection and analysis are available under supplementary information.

### Statistical analysis

Data were presented as mean ± SD. The unpaired *t* test was used for comparisons between groups at each time point. *P* < 0.05 was considered significant.

## Supplementary information

Supplemental Material

## Data Availability

The coordinates for the apo PDE6D alone and bound to Deltarasin or DW0254 have been deposited in the PDB under accession codes 7PAC, 7PAE and 7PAD respectively. Authors will release the atomic coordinates and experimental data upon article publication.
