# Supplementary material for: Validation of a small molecule inhibitor of PDE6D-RAS interaction with favorable anti-leukemic effects
Source: Blood Cancer J. 2022 Apr 14;12(4):64. doi: 10.1038/s41408-022-00663-z (PMC9010429; doi:10.1038/s41408-022-00663-z)
Supplement: Supplementary file 1 — Supplemental Material [file 41408_2022_663_MOESM1_ESM.pdf]

Supplemental Figure 1

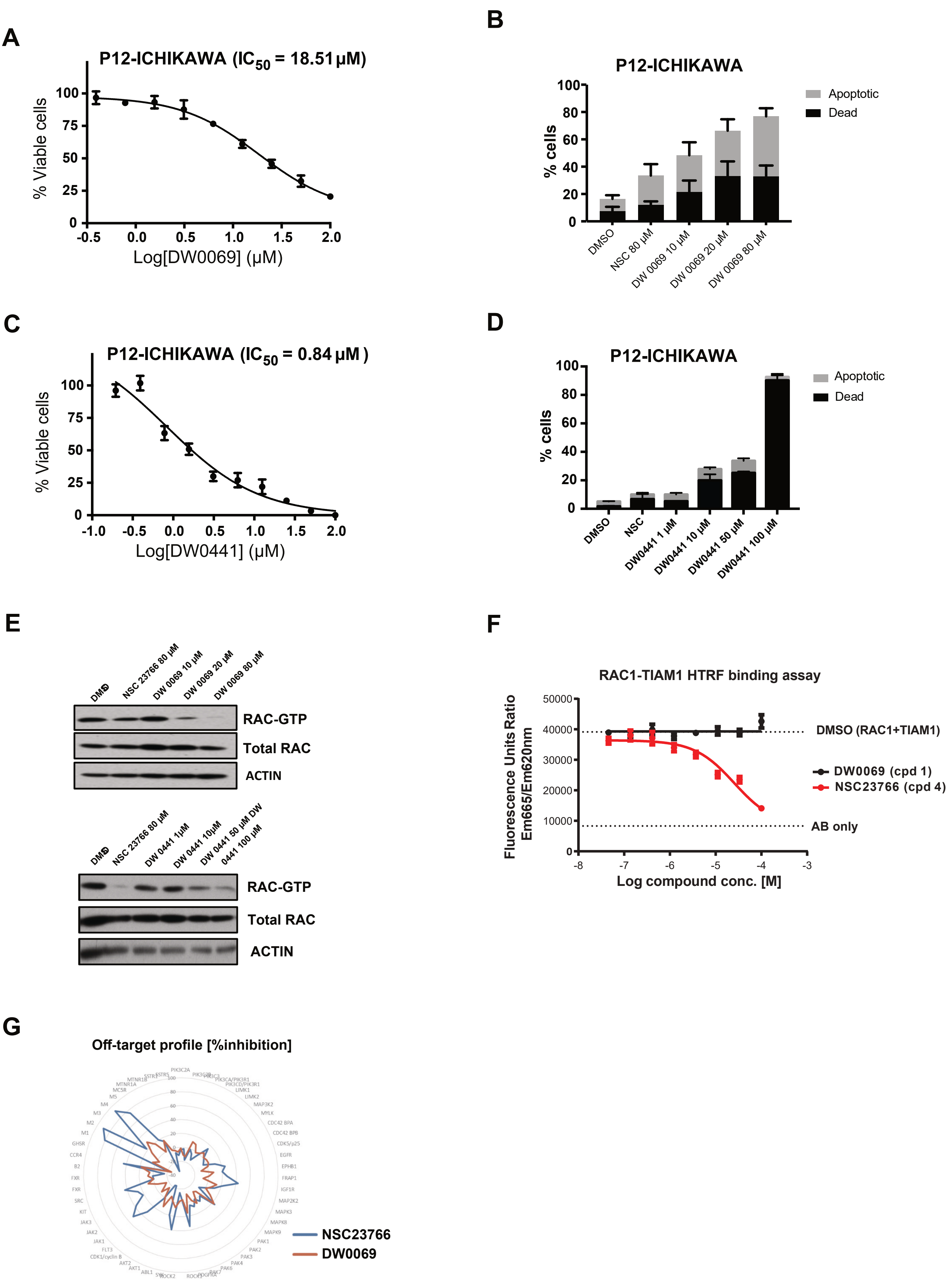

Supplemental Figure 1. DW0069 and DW0441 inhibits RAC activation and show anti-leukemic activity *in vitro*. A) Dose-response curve showing live cell viability assay after 3 days of DW0069 treatment with P12-ICHIKAWA cells. Data represent mean ± SD, n=4 samples for each dose. **B)** Apoptotic and dead P12-ICHIKAWA cells analyzed by Annexin V/PI staining after treatment with different doses of DW0069 compared to the cells treated with 80μM NSC 23766 (NSC) or DMSO control. Apoptotic cells: $AnnV^+/PI^-$, Dead cells: $AnnV^-/PI^+$. Data represent mean ± SD, n=3 samples for each condition. **C)** Dose-response curve showing live cell viability assay after 3 days of DW0441 treatment with P12-ICHIKAWA cells. Data represent mean ± SD, n=3 for each condition. **D)** Apoptotic or dead P12-ICHIKAWA cells treated with different doses of DW0441 compared to the cells treated with 80μM NSC or DMSO control as analyzed by Annexin V/PI staining. Apoptotic cells: $AnnV^+/PI^-$, Dead cells: $AnnV^-/PI^+$. Data represent mean ± SD, n=3 samples for each condition. **E)** Inhibition of RAC activation in SEM cells treated with different doses of DW0069 (upper panel) or P12-ICHIKAWA cells treated with different doses of DW0441 (lower panel). Activated (GTP-bound) RAC was determined by GST pulldown assays as described in Figure 2E. **F)** Compound titration in the RAC1-TIAM1 homogeneous time-resolved fluorescence assay (HTRF) assay showing competition with increasing concentrations of test compounds (either NSC 23766 or DW0069) on X-axis and fluorescence emission (Y-axis). **G)** Radarplot of Off-Target profiling at 20 μM against a focused set of kinases and GPCRs (antagonist mode) for NSC23766 (blue) and DW0069 (red).

Supplemental Figure 2

A

Human PDE6D (UniProtKB - O43924)

msakderare ilrgfklnwm nlrdaetgk**I LWQGTEDLSV PGVEHEAR**vp kkilkckavs r**ELNFSSTEQ MEK**frleqkv
yfkgqcleew ffefgfvipn stntwqslie aapesqmmpa svltgnviie tk**FFDDDLLV STSR**vrlfyv

Sequence coverage: 28,6%

B

```
        Exon1                                                  Exon2
WT      atgtcagccaaggacgagcgggccagggagatcctgaggggcttcaaactaaattggatgaaccttcgggatgctgagacagggaagatactctggcaaggaacagaagacctgt
-6 bp   ATGTCAGCCAAGGACGAGCGGGCCAGGGAGATCCTGAGGGGCTTCAAACTAAATTGGATGAACCTTCGGGATGCTGAGACAGGGAAGATACTCTGGCAAGGAACAGAAGACCTGT
+1 bp   ATGTCAGCCAAGGACGAGCGGGCCAGGGAGATCCTGAGGGGCTTCAAACTAAATTGGATGAACCTTCGGGATGCTGAGACAGGGAAGATACTCTGGCAAGGAACAGAAGACCTGT
-8 bp   ATGTCAGCCAAGGACGAGCGGGCCAGGGAGATCCTGAGGGGCTTCAAACTAAATTGGATGAACCTTCGGGATGCTGAGACAGGGAAGATACTCTGGCAAGGAACAGAAGACCTGT

        Exon2                        Exon3
WT      ctgtccctggtgtggagcatgaagcccgtg ttcccaagaaaatcctcaagtgcaaggcagtgtctcgagaacttaattttcttcgacagaacaaatggaaaaattccgcctgg
-6 bp   CTGTCCCTGGTGTGGAGCATGAAGCCC--- ---CCAAGAAAATCCTCAAGTGCAAGGCAGTGTCTCGAGAACTTAATTTTTCTTCGACAGAACAAATGGAAAAATTCCGCCTGG
+1 bp   CTGTCCCTGGTGTGGAGCATGAAGCCCGTGNTTCCCAAGAAAATCCTCAAGTGCAAGGCAGTGTCTCGAGAACTTAATTTTTCTTCGACAGAACAAATGGAAAAATTCCGCCTGG
-8 bp   CTGTCCCTGGTGTGGAGCATGAAGCCCGTG --------AAAATCCTCAAGTGCAAGGCAGTGTCTCGAGAACTTAATTTTTCTTCGACAGAACAAATGGAAAAATTCCGCCTGG

        Exon3                        Exon4
WT      aacaaaaagtttacttcaaagggcaatgcctagaagaatggttcttcgagtttggctttgtgatccctaactccacaaatacctggcagtccttgatagaggcagcacccgagtc
-6 bp   AACAAAAAGTTTACTTCAAAGGGCAATGCCTAGAAGAATGGTTCTTCGAGTTTGGCTTTGTGATCCCTAACTCCACAAATACCTGGCAGTCCTTGATAGAGGCAGCACCCGAGTC
+1 bp   AACAAAAAGTTTACTTCAAAGGGCAATGCCTAGAAGAATGGTTCTTCGAGTTTGGCTTTGTGATCCCTAACTCCACAAATACCTGGCAGTCCTTGATAGAGGCAGCACCCGAGTC
-8 bp   AACAAAAAGTTTACTTCAAAGGGCAATGCCTAGAAGAATGGTTCTTCGAGTTTGGCTTTGTGATCCCTAACTCCACAAATACCTGGCAGTCCTTGATAGAGGCAGCACCCGAGTC

        Exon4                        Exon5
WT      ccagatgatgccagcaagcgtcttaactgggaacgttatcatagaaacaaagtttttgacgacgatcttcttgtaagcacatccagagtgagacttttctat
-6 bp   CCAGATGATGCCAGCAAGCGTCTTAACTGGGAACGTTATCATAGAAACAAAGTTTTTGACGACGATCTTCTTGTAAGCACATCCAGAGTGAGACTTTTCTAT
+1 bp   CCAGATGATGCCAGCAAGCGTCTTAACTGGGAACGTTATCATAGAAACAAAGTTTTTGACGACGATCTTCTTGTAAGCACATCCAGAGTGAGACTTTTCTAT
-8 bp   CCAGATGATGCCAGCAAGCGTCTTAACTGGGAACGTTATCATAGAAACAAAGTTTTTGACGACGATCTTCTTGTAAGCACATCCAGAGTGAGACTTTTCTAT
```

C

```
WT      MSAKDERAREILRGFKLNWMNLRDAETGKILWQGTEDLSVPGVEHEARV--PKKILKCKAVSRELNFSSTEQMEKFRLEQKVYFKGQCLEEWFFEFGFVIPNSTNTWQSL 108
-6 bp   MSAKDERAREILRGFKLNWMNLRDAETGKILWQGTEDLSVPGVEHEAK-----KILKCKAVSRELNFSSTEQMEKFRLEQKVYFKGQCLEEWFFEFGFVIPNSTNTWQSL 105
+1 bp   --------------------MLRQGR-----YSGKEQKTCLSLVWSMKPVXPKKILKCKAVSRELNFSSTEQMEKFRLEQKVYFKGQCLEEWFFEFGFVIPNSTNTWQSL 85
-8 bp   --------------------MLRQGR-----YSGKEQKTCLSLVWSMK---PVKILKCKAVSRELNFSSTEQMEKFRLEQKVYFKGQCLEEWFFEFGFVIPNSTNTWQSL 82

WT      IEAAPESQMMPASVLTGNVIIETKFFDDDLLVSTSRVRLFYV 150
-6 bp   IEAAPESQMMPASVLTGNVIIETKFFDDDLLVSTSRVRLFYV 147
+1 bp   IEAAPESQMMPASVLTGNVIIETKFFDDDLLVSTSRVRLFYV 127
-8 bp   IEAAPESQMMPASVLTGNVIIETKFFDDDLLVSTSRVRLFYV 124
```

Supplemental Figure 2. Edits caused by sgRNA#144 in DW0254 resistant cells. A) PDE6D sequence coverage by LC-MS/MS after PAL pulldown in P12-ICHIKAWA cells. **B)** cDNA sequences of WT PDE6D aligned with -6bp, +1bp and -8bp mutations found in sgRNA#144 transduced cells after long term treatment (21 days) with DW0254, different highlight colors on the cDNA sequence represent the different exons. Eddits are highlighted in red (deletions) or yellow (insertions). **C)** WT PDE6D amino acid sequence aligned with the resulting protein sequences of -6bp, +1bp and -8bp mutations found in sgRNA#144 transduced cells after long term (21 days) treatment with DW0254.

Supplemental Figure 3

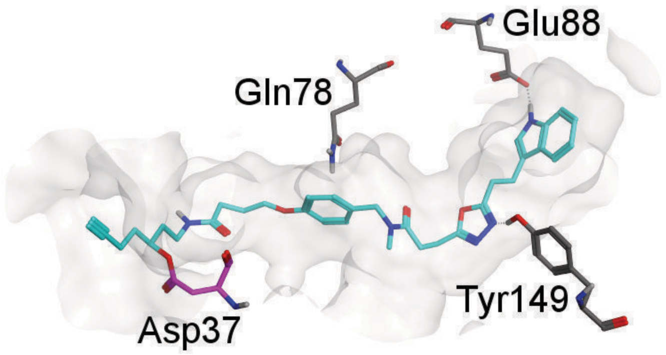

Supplemental Figure 3. Docking pose of PAL on PDE6D hydrophobic pocket. Docking protocol recapitulates PALMS assay results and shows a covalent bond formed with D37 in PDE6D (view rotated from Fig 4B).

Supplemental Figure 4

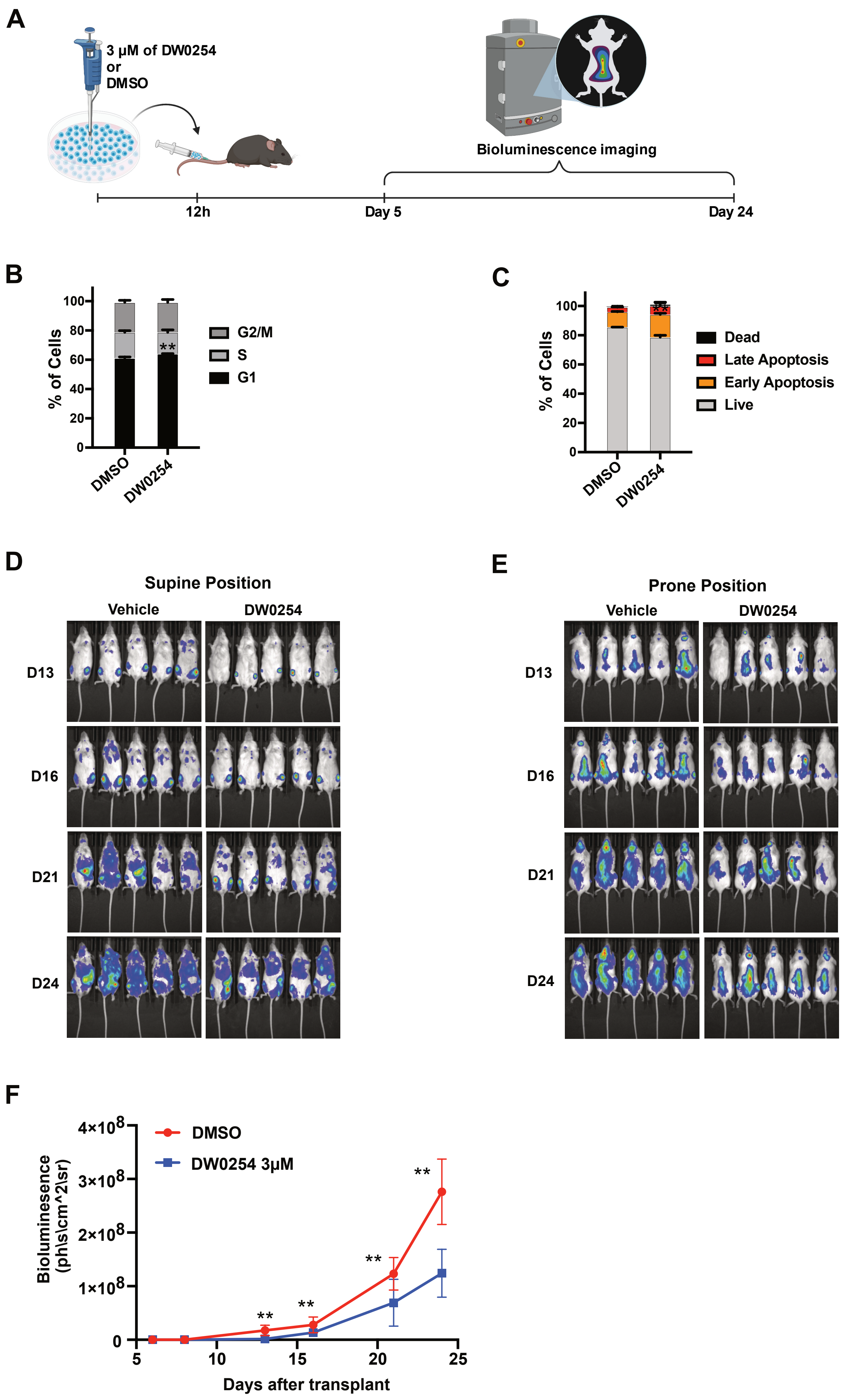

Supplemental Figure 4. DW0254 *ex vivo* treatment reduces leukemic tumor burden of P12-ICHIKAWA mouse xenograft model. A) Schedule of *ex vivo* treatment. Luciferase expressing P12-ICHIKAWA cells treated for 12 hours with either 3μM of DW0254 or DMSO before transplantation into NSG mice; Bioluminescence imaging was performed every 3-5 days to assess tumor burden between day 5 and day 24 after transplantation. Created with BioRender.com. **B)** Bar graph showing the cell cycle distribution by DAPI staining of P12-ICHIKAWA cells treated for 12 hours with 3μM of DW0254, data represent mean ± SD of 2 independent experiments with n=3 samples for each condition, ** p<0.01. **C)** Bar graph showing percentage of apoptosis by AnnV/DAPI staining of P12-ICHIKAWA cells treated for 12 hours with 3μM of DW0254, data represent mean ± SD of 2 independent experiments with n=3 samples for each condition, ** p<0.01. Live: $AnnV^-/DAPI^-$; Early apoptosis: $AnnV^+/DAPI^-$; Late apoptosis: $AnnV^+/DAPI^+$; and Dead: $AnnV^-/DAPI^+$. **D)** Representative images in the prone or **E)** supine position of bioluminescence imaging (BLI) from NSG mice transplanted with P12-ICHIKAWA leukemia cells expressing luciferase after treatment with 3μM DW0254 *ex vivo* for 12 hours. **F)** Quantification of BLI in NSG mice transplanted with luciferase-expressing P12-ICHIKAWA cells after *ex vivo* treatment with 3μM DW0254 for 12 hours. The days after transplantation are shown in X-axis, and the bioluminescence intensity is shown in Y-axis. Data represents mean ±SD, **p<0.01, n=10 mice per group, and were representative of 2 independent experiments.

Supplemental Table 1. Design strategy of PDE6D sgRNA library and controls

	sgRNA ID	sgRNA Sequence	Orientation	PAM Sequence	Exon Number
PDE6D sgRNA library	sg_hPDE6D_6	CCGCCGCATCATGTCAGCCA	sense	AGG	1
	sg_hPDE6D_12	CTCCCTGGCCCGCTCGTCCT	antisense	TGG	1
	sg_hPDE6D_14	TCATGTCAGCCAAGGACGAG	sense	CGG	1
	sg_hPDE6D_15	CATGTCAGCCAAGGACGAGC	sense	GGG	1
	sg_hPDE6D_20	CAGCCAAGGACGAGCGGGCC	sense	AGG	1
	sg_hPDE6D_21	AGCCAAGGACGAGCGGGCCA	sense	GGG	1
	sg_hPDE6D_27	GAAGCCCCTCAGGATCTCCC	antisense	TGG	1
	sg_hPDE6D_32	AGCGGGCCAGGGAGATCCTG	sense	AGG	1
	sg_hPDE6D_33	GCGGGCCAGGGAGATCCTGA	sense	GGG	1
	sg_hPDE6D_34	CGGGCCAGGGAGATCCTGAG	sense	GGG	1
	sg_hPDE6D_37	GATACAGTTTGAAGCCCCTC	antisense	AGG	1
	sg_hPDE6D_62	TCAGAAATTGGATGAACCTT	sense	CGG	2
	sg_hPDE6D_63	CAGAAATTGGATGAACCTTC	sense	GGG	2
	sg_hPDE6D_67	TCCCTGTCTCAGCATCCCGA	antisense	AGG	2
	sg_hPDE6D_76	AACCTTCGGGATGCTGAGAC	sense	AGG	2
	sg_hPDE6D_77	ACCTTCGGGATGCTGAGACA	sense	GGG	2
	sg_hPDE6D_89	CTGAGACAGGGAAGATACTC	sense	TGG	2
	sg_hPDE6D_94	ACAGGGAAGATACTCTGGCA	sense	AGG	2
	sg_hPDE6D_115	GCTCCACACCAGGGACAGAC	antisense	AGG	2
	sg_hPDE6D_118	ACAGAAGACCTGTCTGTCCC	sense	TGG	2
	sg_hPDE6D_123	AGACCTGTCTGTCCCTGGTG	sense	TGG	2
	sg_hPDE6D_124	TACCTTCATGCTCCACACCA	antisense	GGG	2
	sg_hPDE6D_125	GTACCTTCATGCTCCACACC	antisense	AGG	2
	sg_hPDE6D_133	GTCCCTGGTGTGGAGCATGA	sense	AGG	2
	sg_hPDE6D_140	ATTTTCTTGGGAACACGGGC	antisense	TGG	3
	sg_hPDE6D_144	GAGGATTTTCTTGGGAACAC	antisense	GGG	3
	sg_hPDE6D_145	TGAGGATTTTCTTGGGAACA	antisense	CGG	3
	sg_hPDE6D_152	TTGCACTTGAGGATTTTCTT	antisense	GGG	3
	sg_hPDE6D_153	CTTGCACTTGAGGATTTTCT	antisense	TGG	3
	sg_hPDE6D_163	GAGACACTGCCTTGCACTTG	antisense	AGG	3
	sg_hPDE6D_165	CAAGAAAATCCTCAAGTGCA	sense	AGG	3
	sg_hPDE6D_207	TTTTTCTTCGACAGAACAAA	sense	TGG	3
	sg_hPDE6D_222	ACAAATGGAAAAATTCCGCC	sense	TGG	3
	sg_hPDE6D_226	AGTAAACTTTTTGTTCCAGG	antisense	CGG	3
	sg_hPDE6D_229	TGAAGTAAACTTTTTGTTCC	antisense	AGG	3
	sg_hPDE6D_244	GAACAAAAAGTTTACTTCAA	sense	AGG	3
	sg_hPDE6D_245	AACAAAAAGTTTACTTCAAA	sense	GGG	3
	sg_hPDE6D_259	TTCAAAGGGCAATGCCTAGA	sense	AGG	3
	sg_hPDE6D_262	TAGGCAGCAGAATACCTTCT	antisense	AGG	3
	sg_hPDE6D_277	TCAGAATGGTTCTTCGAGTT	sense	TGG	4
	sg_hPDE6D_298	GCCAGGTATTTGTGGAGTTA	antisense	GGG	4
	sg_hPDE6D_299	TGCCAGGTATTTGTGGAGTT	antisense	AGG	4
	sg_hPDE6D_306	CAAGGACTGCCAGGTATTTG	antisense	TGG	4
	sg_hPDE6D_308	TCCCTAACTCCACAAATACC	sense	TGG	4
	sg_hPDE6D_315	TGCCTCTATCAAGGACTGCC	antisense	AGG	4
	sg_hPDE6D_324	TACCTGGCAGTCCTTGATAG	sense	AGG	4
	sg_hPDE6D_324	CTCGGGTGCTGCCTCTATCA	antisense	AGG	4
	sg_hPDE6D_341	GCTGGCATCATCTGGGACTC	antisense	GGG	4
	sg_hPDE6D_342	TGCTGGCATCATCTGGGACT	antisense	CGG	4
	sg_hPDE6D_348	GACGCTTGCTGGCATCATCT	antisense	GGG	4
	sg_hPDE6D_349	AGACGCTTGCTGGCATCATC	antisense	TGG	4
	sg_hPDE6D_359	ACTCACGTTAAGACGCTTGC	antisense	TGG	4
	sg_hPDE6D_432	AACATAGAAAAGTCTCACTC	antisense	TGG	5
Negative Control sgRNAs	sg_Neg_1	GTAGCGAACGTGTCCGGCGT			
	sg_Neg_2	GACCGGAACGATCTCGCGTA			
	sg_Neg_3	GGCAGTCGTTCGGTTGATAT			
	sg_Neg_4	GCTTGAGCACATACGCGAAT			
	sg_Neg_5	GTGGTAGAATAACGTATTAC			
	sg_Neg_6	GTCATACATGGATAAGGCTA			

		sgRNA ID	sgRNA Sequence
Negative Control sgRNAs		sg_Neg_7	GATACACGAAGCATCACTAG
		sg_Neg_8	GAACGTTGGCACTACTTCAC
		sg_Neg_9	GATCCATGTAATGCGTTCGA
		sg_Neg_10	GTCGTGAAGTGCATTCGATC
		sg_LacZ_1465	CATCGGGCAAATAATATCGG
		sg_LacZ_1583	TCGCGTGGGCGTATTCGCAA
		sg_LacZ_1659	ACGAAGCCGCCCTGTAAACG
		sg_LacZ_1725	ATGATGAAAACGGCAACCCG
		sg_LacZ_1739	AACCCGTGGTCGGCTTACGG
		sg_LacZ_2496	TGATTACGACCGCTCACGCG
		sg_LacZ_336	GTGAGCGAGTAACAACCCGT
		sg_LacZ_838	CAGACGTAGTGTGACGCGAT
		sg_LacZ_904	AATCCCGAATCTCTATCGTG
		sg_LacZ_907	CCCGAATCTCTATCGTGCGG
		sg_Luc_1058	AGCTATTCTGATTACACCCG
		sg_Luc_1158	ACGCTGGGCGTTAATCAAAG
		sg_Luc_1189	ACATAACCGGACATAATCAT
		sg_Luc_1451	GTGCTCCAAAACAACAACGG
		sg_Luc_1536	ACAACCGCGAAAAAGTTGCG
		sg_Luc_1539	ACCGCGAAAAAGTTGCGCGG
		sg_Luc_371	GGGCATTTCGCAGCCTACCG
		sg_Luc_474	GATTCTAAAACGGATTACCA
		sg_Luc_560	TGTGCCAGAGTCCTTCGATA
		sg_Luc_650	GGCATGCGAGAATCTCACGC
		sg_Ren_158	TATTTTTTTACATGGTAACG
		sg_Ren_170	ATGTCGCCATAAATAAGAAG
		sg_Ren_211	GGTATAATACACCGCGCTAC
		sg_Ren_307	ATTACAAATATCTTACTGCA
		sg_Ren_55	GGATGATAACTGGTCCGCAG
		sg_Ren_58	TTACATCTGGCCCACCACTG
		sg_Ren_649	TCACGAGGCCATGATAATGT
		sg_Ren_664	TTTACTAACGGGATTTCACG
		sg_Ren_681	GAAATCCCGTTAGTAAAAGG
		sg_Ren_794	GGCACCTTCAACAATAGCAT
		sg_ROSA26_1	GAAGATGGGCGGGAGTCTTC
Positive Control sgRNAs		sg_PCNA_1	GGACTCGTCCCACGTCTCTT
		sg_PCNA_2	CTACCGCTGCGACCGCAACC
		sg_POLR2D_1	TGAGAGTGCAGAGGACGAAC
		sg_POLR2D_2	TGGGCAAAGGTTGGCCAAAC
		sg_POLR2A_1	AAGCGAATGTCTGTGACGGA
		sg_POLR2A_2	CAGGGGGTGATTGAGCGGAC
		sg_PRL9_1	GGACGCACAGTTATCGTGAA
		sg_PRL9_2	AATGTAGAACTCAGCCTTCT
		sg_RPL23A_1	TGCGGATCTTCTTCTTTTTG
		sg_RPL23A_2	GTCGCAGTGTCTTCGGCCGC
		sg_CDK9_1	CTGGCCGGTCTTGCGGTGCC
		sg_CDK9_2	GATCTCCCGCAAGGCTGTAA
		sg_CDK1_1	GGGTTCCTAGTACTGCAATT
		sg_CDK1_2	AATCCATGTACTGACCAGGA
		sg_RPA3_1	CCGGCGTTGATGCGCGACCT
		sg_RPA3_2	GCCGGCGTTGATGCGCGACC
		sg_hBRD4_309	TAAGATCATTAAAACGCCTA
		sg_hBRD4_336	GGGAACAATAAAGAAGCGCT
		sg_hMYC_1138	ACAACGTCTTGGAGCGCCAG
		sg_hMYC_122	GCCGTATTTCTACTGCGACG
		sg_hRPS20_172	AAGCCGCAACGTAAAATCCT
		sg_hRPS20_90	ACCAGTTCGAATGCCTACCA

Supplemental Methods:

PAL Probe Synthesis

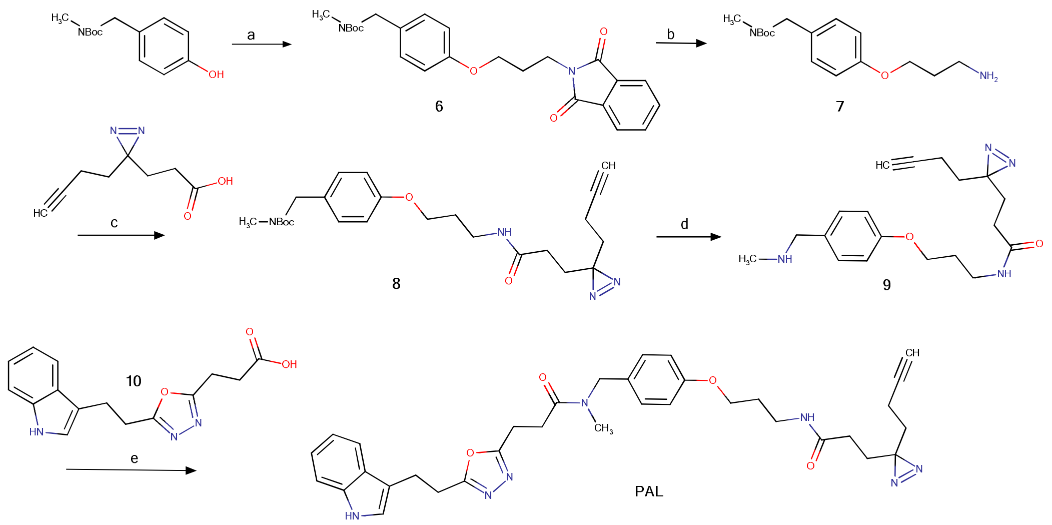

Reagents and conditions: a) N-(3-bromopropyl)phtalimide, Sodium iodide, Cesium carbonate, DMF; b) Hydrazine Hydrate, EtOH; c) HATU, N-diisopropyethylamine, DCM; d) TFA, DCM; e) HATU, N-diisopropyethylamine, DMF.

All chemicals were purchased from commercial suppliers and used as received unless otherwise specified. NMR spectra were recorded at 300K on 600MHz NMR spectrometers Bruker AVANCE III equipped with TCI 5mm cryoprobe and on 500MHz Bruker AVANCE I equipped with BBI 5mm probe. All samples were dissolved in 0.6 ml of DMSO-d6. Chemical shifts are reported in ppm (δ) referenced to TMS (δ = 0.00 ppm) and DMSO (2.50 ppm). LC−MS analysis was performed on a Waters Acquity UPLC analytical system with DAD (UV detection at 220nm) and a single quadrupole mass spectrometer (QDa Detector) equipped with an ACQUITY UPLC CSH C18 column, 2.1 mm × 50 mm, 1.7 µm. Method: ESI+, Flow rate 0.8 mL/min, Eluent A : H2O + 0.05% TFA, Eluent B : CH3CN+0.035% TFA, Gradient : from 2% to 98% of B in 2.4min, hold 0.6min. Intermediates 6-10 were used without full characterization.

The syntheses of compounds 1-5 and intermediate 10 were performed according to methods described in WO2019/143833.

5- tert-butyl N-[[4-(3-aminopropoxy)phenyl]methyl]-N-methyl-carbamate (6).

To a stirred solution of tert-butyl N-[(4-hydroxyphenyl)methyl]-N-methylcarbamate (0.665 g, 2.80 mmol) in anhydrous DMF (27 ml) under nitrogen were added NaI (0.281 g, 1.88 mmol), Cs2CO3 (2.74 g, 8.41 mmol) and N-(3-bromopropyl)phtalimide (0.976 g, 3.64 mmol). The reaction mixture was stirred at 40°C for 30 min, quenched with water and extracted with ethyl acetate. The combined organic layers were dried over Na2SO4, and concentrated. The crude mixture was purified by flash chromatography on silica gel using dichloromethane to afford 6 (1.16 g, 97%) as a colorless oil. 1H NMR (DMSO-d6, 500 MHz) δ 7.90-7.80 (m, 4H), 7.09 (d, 2H, J=8.6 Hz), 6.77 (d, 2H, J=8.6 Hz), 4.26 (s, 2H), 3.99 (t, 2H, J=5.9 Hz), 3.76 (t, 2H, J=6.8 Hz), 2.70 (s, 3H), 2.05 (m, 2H), 1.41 (br s, 9H). LRMS (m/z) [M+Na]+ 447.1

tert-butyl-N-[[4-(3-aminopropoxy)phenyl]methyl]-N-methyl-carbamate (7). To a stirred solution of 6 (1.11 g, 2.62 mmol) in ethanol (16 ml) under nitrogen was added hydrazine hydrate (0.38 ml, 7.85 mmol). The reaction mixture was stirred at reflux for 1 h, then cooled to rt, filtered and the filtrate was concentrated. The resulting residue was dissolved in ethyl acetate, washed with water, dried over sodium sulfate, and concentrated to afford 7 (0.720 g, 84% yield) as colourless oil. 1H NMR (DMSO-d6, 500 MHz) δ 7.13 (br d, 2H, J=8.3 Hz), 6.90 (br d, 2H, J=8.3 Hz), 4.28 (s, 2H), 4.00 (t, 2H, J=6.3 Hz), 2.71 (s, 3H), 2.68 (t, 2H, J=6.6 Hz), 1.76 (m, 2H), 1.67 (br s, 2H), 1.41 (br s, 9H). LRMS (m/z) [M+H]+ 295.1.

tert-butyl-N-[[4-[3-[3-(3-but-3-ynyldiazirin-3-yl)propanoylamino]propoxy] phenyl]methyl]-N-methyl-carbamate (8) To a stirred solution of 7 (0.089 g, 0.27 mmol), 3-(3-but-3-ynyldiazirin-3-yl)propanoic acid (0.050 mg, 0.30 mmol, Li et al, 2013) and HATU (0.124 g, 0.32 mmol) in dichloromethane (4.3 ml) in the dark under nitrogen was added N-diisopropylethylamine (0.118 ml, 0.68 mmol). The reaction

mixture was stirred at rt overnight, then diluted with dichloromethane, washed with saturated aqueous solution of sodium carbonate, brine, dried over Na2SO4, and concentrated to afford 8 (0.152 g, 100%) as brown oil. 1H NMR (DMSO-d6, 600 MHz) δ 7.90 (br t, 1H, J=5.4 Hz), 7.13 (d, 2H, J=8.5 Hz), 6.89 (br d, 2H, J=8.5 Hz), 4.28 (s, 2H), 3.96 (t, 2H, J=6.3 Hz), 3.21-3.15 (m, 2H), 2.80 (t, 1H, J=2.6 Hz), 2.70 (s, 3H), 1.97 (dt, 2H, J=2.6, 7.5 Hz), 1.90-1.86 (m, 2H), 1.82 (quin, 2H, J=6.6 Hz), 1.66-1.62 (m, 2H), 1.55 (t, 2H, J=7.3 Hz), 1.41 (br s, 9H). LRMS (m/z) [M+H]+ 443.1.

3-(3-but-3-ynyldiazirin-3-yl)-N-[3-[4-(methylaminomethyl)phenoxy]propyl] propanamide (9). Deprotection of 8 (0.141 g, 0.250 mmol) was achieved in 1h using trifluoroacetic acid (0.280 ml, 3.63 mmol) in dichloromethane (0.280 ml) whilst stirring at 0-5°C in the dark. The mixture was neutralized using 2M cold aqueous sodium carbonate solution. The aqueous layer was extracted with DCM. The combined organic layers were washed with brine, dried over Na2SO4, and concentrated to afford compound 9 (0.122 g, 100%) as brown oil (quantitative yield). 1H NMR (DMSO-d6, 500 MHz) δ 7.91 (t, 1H, J=6.0 Hz), 7.23-7.18(m, 2H, J=8.6 Hz), 6.87-6.83 (m, 2H, J=8.6 Hz), 3.95 (t, 2H, J=6.4 Hz), 3.57 (s, 2H), 3.33 (br s, 1H), 3.18 (q, 2H, J=6.6 Hz), 2.81 (t, 1H, J=2.6 Hz), 2.24 (s, 3H), 1.98 (dt, 2H, J=2.7, 7.3 Hz), 1.91-1.86 (m, 2H), 1.86-1.80 (m, 2H), 1.64 (t, 2H, J=7.6 Hz), 1.56 (t, 2H, J=7.3 Hz). LRMS (m/z) [M+H]+ 343.1.

Synthesis of N-[[4-[3-[3-(3-but-3-ynyldiazirin-3-yl)propanoylamino]propoxy]phenyl] methyl]-3-[5-[2-(1H-indol-3-yl)ethyl]-1,3,4-oxadiazol-2-yl]-N-methyl-propanamide (PAL). To a stirred solution of 3-[5-[2-(1H-indol-3-yl)ethyl]-1,3,4-oxadiazol-2-yl]propanoic acid 10 (0.076 g, 0.270 mmol) in anhydrous N,N-dimethylformamide (1.90 ml) was added N-ethyldiisopropylamine (0.230 ml, 1.34 mmol), HATU (0.153 g, 0.400 mmol) and 9 (0.102 g, 0.270 mmol). The reaction mixture was stirred in the dark at rt for 4 h, poured in cold water and extracted with ethyl acetate. The combined organic layers were washed with water, dried over Na2SO4, filtered and

concentrated. The crude was purified by flash chromatography on silica gel using dichloromethane: methanol (1-4%) to afford PAL (0.105 g) as light brown gum (64% yield). 1H NMR (DMSO-d6, 600 MHz) (2 conformers 65/35 at 300K) δ 10.80 (br s, 1H), 7.90 (t, 1H, J=5.3 Hz), 7.50-7.46 (m, 1H), 7.34-7.30 (m, 1H), 7.16-7.11 (m, 3H), 7.08-7.03 (m, 1H), 7.00-6.94 (m, 1H), 6.93-6.83 (m, 2H), 4.50 (s, 0.35H), 4.42 (s, 0.65H), 3.97-3.91 (m, 2H), 3.20-3.09 (m, 6H), 3.05-2.99 (m, 2H), 2.89 (s, 2H), 2.87-2.79 (m, 3H), 2.78 (s, 1H), 1.97 (dt, 2H, J= 2.6, 7.6 Hz), 1.88 (t, 2H, J=7.6 Hz), 1.84-1.78 (m, 2H), 1.64 (dd, 2H, J=6.9, 8.4 Hz), 1.55 (t, 2H, J=7.6Hz). 13C NMR (DMSO-d6, 151 MHz)2 conformers at RT δ 171.1, 170.6, 170.5, 166.6, 166.6, 166.5, 166.4, 158.3, 158.1, 136.6, 130.0, 129.4, 129.4, 128.4, 127.3, 123.2, 123.2, 121.4, 118.8, 118.5, 115.1, 114.8, 112.9, 111.9, 83.6, 72.2, 65.6, 65.6, 52.1, 49.8, 40.5, 36.0, 34.7, 33.7, 31.9, 29.9, 29.3, 29.2, 29.0, 28.7, 28.5, 26.3, 26.2, 22.3, 22.3, 21.1, 21.0, 13.1. High-resolution accurate mass measurement gives the pseudo molecular ion MH+ at m/z 610,3418 which is in agreement with the proposed elemental formula (C34H39N7O4 + H+: 610,3142). Mass measurement was obtained with a precision of 0,5 ppm.

Intact Cell-Based Photoaffinity Labelling with PAL Probe and LC-MS/MS analysis

In situ competitive photoaffinity labelling

For in situ labelling, 5 x 106 P12-ICHIKAWA or CCRF-CEM cells were resuspended in PBS and treated in the dark with 1 μM PAL or DMSO during 30 min at 37°C. For competitive photolabelling experiments, a 15 min-pretreatment with 20 μM DW0254 was followed by PAL treatment during 30 min. For each condition, three biological replicates were prepared. After treatment, cells were washed with cold PBS and UV-irradiated (365 nm) using an UVP CL-1000 UV crosslinking chamber (Thermo Fischer Scientific) in PBS for 20 min at 4 °C to activate the diazirine for probe–target covalent labelling. After UV-irradiation, cells were pelleted by

centrifugation. The cell pellets were resuspended in SDS lysis buffer containing 1% w/v SDS in 50 mM Tris-HCl, pH 8.0, with Roche protease inhibitor tablet (Sigma-Aldrich) and 1% v/v Benzonase® Nuclease, Purity >99% (Millipore) and vortex-mixed for 30 min at 37°C. Lysates were clarified by centrifugation and protein amounts were measured using the Pierce BCA Protein Assay Kit (Thermo Fisher Scientific). Lysates were reduced with 10 mM dithiothreitol (DTT) for 1 h at room temperature (RT) and then alkylated with 30 mM iodoacetamide for 45 min at RT in the dark. After reduction and alkylation, protein lysates were precipitated with 9 volumes prechilled acetone for 18 h at -20 °C. Precipitated proteins were subsequently collected by centrifugation (16,000 × g for 10 min at 4 °C). The supernatants were discarded, and the protein pellets were washed with prechilled acetone (500 µL) and precipitated by centrifugation. After removing the supernatants, the protein pellets were air-dried and dissolved in SDS lysis buffer by vortexing thoroughly. Sample protein concentrations were adjusted to 3 mg/mL with SDS lysis buffer. For affinity-capture of PAL photo-crosslinked proteins with streptavidin-coated beads, cell lysate proteins (200 µg) of each replicate were labelled with 100 µM Biotin Azide (PEG4 carboxamide-6-Azidohexanyl Biotin) probe using the Click-iTTM Protein Reaction Buffer Kit (all Thermo Fisher Scientific) then precipitated by adding acetone. Biotin-labelled protein pellets were resuspended in 60 µL SDS lysis buffer then adjusted to 1.5 mL with the binding buffer containing 50 mM Tris-HCl, pH 7.5, 150 mM NaCl and protease inhibitors (0.04% w/v SDS, final concentration) and kept at 4°C until use. Enrichment of Photo-Crosslinked Proteins for LC-MS/MS Analysis

Streptavidin Mag Sepharose® magnetic beads (GE Healthcare) (60 µL of a 50% v/v slurry was used per sample) were washed thoroughly with the binding buffer. The beads were then incubated with biotin-labelled proteins (200 µg) resuspended in the binding buffer for 3h at RT with gentle rotation. After binding, the beads were washed twice with 50 mM Tris, 150 mM NaCl pH 7.5, 2 M urea followed by two washed with 50 mM NH4HCO3. The beads were then resuspended in 50 mM

NH4HCO3 and kept at 4°C until use. The beads were used either for on-bead protein digestion (P12-ICHIKAWA biotin-labelled proteins) or in-gel protein digestion (CCRF-CEM biotin-labelled proteins) followed by label-free LC-MS/MS quantitative analysis.

Sample Preparation for LC-MS/MS Analysis

For on-bead digestion, P12-ICHIKAWA biotin-labelled proteins bound onto streptavidin beads were digested with 1 µg of Trypsin/LysineC mix, (Promega) to a final ratio enzyme:substrate of 1:25 (w/w) and incubated overnight at 37°C under mild agitation. The supernatants containing the resulting peptides were collected. and acidified with FA (0.2 %, final concentration), and filtered using Costar® Spin-X® Centrifuge Tube Filters, 0.22 µm Pore Cellulose Acetate Membrane (Fisher Scientific). Four microliters of iRT peptides 1X (Biognosis) were added and sample volumes were finally adjusted to 100 µL with 0.2% FA.

For in-gel digestion, CCRF-CEM biotin-labelled proteins bound onto streptavidin beads were mixed with 50 µL XT Sample Buffer 1X containing 2.5% v/v 2-mercaptoethanol and 30 mM biotin and heated (95 °C, 15 min) to release the proteins from the beads. Protein eluted from the beads (50 µL per well) were resolved using SDS-PAGE and the gel was stained with the PierceTM PageBlueTM protein staining solution (Thermo Fisher Scientific). For each condition tested, three protein bands around 17 kDa corresponding to PDE6D molecular weight were cut out from the gel and washed with 50 mM of NH4HCO3 and ACN (1:1) until Coomassie blue is removed. Thereafter, the gel pieces were treated at 56°C for 30 min with 10 mM of DTT in 50 mM of NH4HCO3 and washed with 50 mM NH4HCO3 and ACN (1:1). The proteins were subsequently alkylated with 55 mM of iodoacetamide in 50 mM of NH4HCO3 for 30 min and washed with 50 mM NH4HCO3 and ACN (1:1). Then, gel pieces were resuspended in 50 mM of NH4HCO3 and digested overnight with Trypsin/LysineC Mix at 0.2 µg/µl (3 µg) at 37°C. The resulting peptides were extracted from the gel with three successive buffers as follow: 1) 50 % ACN, 0.2 % FA 2) 75 % ACN, 0.2 % FA 3) ACN/ethanol (1:1). The extracted peptides were

pooled and combined with the initial digestion supernatant and dried in a SpeedVac. Peptide mixtures were reconstituted with four microliters of iRT peptides 1X and sample volumes were finally adjusted to 100 µL of 50 mM NH4HCO3/0.2% FA.

LC-MS/MS Analysis of Peptide Mixtures

The peptides were analyzed by nanoLC-MS/MS using an Ultimate 3500 RSLC System (Dionex) coupled to a Q-Exactive Plus mass spectrometer (Thermo Fisher Scientific) Twenty µl of each sample were loaded onto a C18 precolumn Acclaim™ PepMap™ C18, 100 Å, 5 µm, 300 µm ID x 5 mm (Thermo Fisher Scientific) at 4°C at 20 µl/min in 2 % ACN, 0.05 % trifluoroacetic acid (TFA). After 3 min of desalting, the precolumn was switched online with the analytical C18 column Acclaim™ PepMap™ RSLC C18, 100 Å, 2 µm, 75 µm ID x 15 cm (Thermo Fisher Scientific) equilibrated with 92 % solvent A (0.2 % FA) and 8 % solvent B (80 % ACN, 0.2 % FA). The peptides were eluted using a 8 to 41 % gradient of solvent B during 120 min at 300 nL/min flow rate. The mass spectrometer was operated in the data-dependent mode. Survey full scan MS spectra (from m/z 325-1300) were acquired with a resolution of 70,000 at m/z 200. The AGC was set to 3 × 106 with a maximum injection time of 30 ms. The top 10 most intense ions (5≥ z ≥2) were targeted for fragmentation (AGC of 1 × 105 and a maximum injection time of 80 ms for an intensity threshold of 4 × 104). Isolation windows at 2 m/z. The dynamic exclusion time window was set to 50 s to prevent repetitive selection of the same peptide. MS2 spectra were recorded in profile type with a resolution of 17,500. An exclusion list of 11 streptavidin peptides was activated to reduce the sequencing time of streptavidin peptides. Injection duplicates were performed for each sample.

In-vitro Photolabeling of PDE6D with PAL Probe and LC-MS/MS Analysis

In vitro competitive photoaffinity labelling

Recombinant human His-TEV-PDE6D-Avitag protein (1 μg, 47 pmol, final concentration 1 μM) was separately incubated for 5 min in PBS with 2.5 μM of PAL or DMSO (final reaction volume, 50 μL). After incubating in the dark at RT for 30 min, the mixtures were UV-irradiated as previously. For competitive photoaffinity labelling experiments, a 5-min pretreatment with 25 μM DW0254 was followed by the treatment with 2.5 μM of PAL or DMSO and photolysis. After UV-irradiation, 1% SDS and 10 mM DTT were added, and after incubation for 1 h at 56°C, protein samples were treated with 30 mM iodoacetamide for 45 min at RT in the dark. After acetone precipitation, the precipitated protein pellets were air-dried for 10 min at RT and resuspended in 30 μL of 1% SDS in 50 mM Tris-HCl, pH 7.5. Probe-labelled PDE6D was tagged with 100 μM tetramethylrhodamine (TAMRA) azide (Thermo Fisher Scientific) by copper click chemistry according to the manufacturer's instructions. Proteins were then precipitated using the chloroform-methanol method described by Wessel and Flügge (Wessel and Flügge, 1984) and the precipitated protein pellets were air-dried for 10 min at room temperature.

Preparation of Labelled PDE6D for MS-Analysis

Recombinant human His-TEV-PDE6D-Avitag protein (50 μM, 2.5 nmol) in 100 μL PBS was preincubated with 50 μM of DW0254 or DMSO for 5 min and then treated with 50 μM PAL for another 5 min at RT. The samples were UV-irradiated as previously. After photolysis, 50 μL urea 8 M was added (4 M urea final concentration). The samples were reduced with 10 mM DTT in 50 mM NH4HCO3 at 56°C for 30 min and alkylated with 55 mM iodoacetamide for 30 min at RT in the dark. After buffer exchange, Trypsin/LysC Mix (1:25 w/w ratio enzyme:protein) was added to the denatured protein diluted in 100 mM sodium phosphate pH 7.8. The mixture was incubated at 37°C overnight. Peptide mixtures (10 μg) were cleaned-up using Bond Elut OMIX Pipette-based SPE C18 tips (Agilent) and resuspended in 10 μL of 0.2 % FA/5% DMSO until use. LC-MS/MS Analysis of PDE6D Peptides

Peptides were analyzed by nanoLC-MS/MS with the Ultimate 3000 RSLC (Thermo Fisher Scientific) coupled online to a Q-Exactive Plus mass spectrometer with an analytical column (40 cm long, 75 µm ID, C18-AQ, 1.9 µm). The peptide mixture (5 µl) was loaded onto the analytical column with 5% solvent B (80% ACN, 5% DMSO, 0.2% FA) in solvent A (5% DMSO, 0.2% FA) at a flow rate of 400 nL/min and separated with a linear gradient of 5% to 30% solvent B at a flow rate of 300 nL/min over 103 min. The Q-Exactive Plus was operated in data-dependent acquisition mode using the following settings: full-scan automatic gain control (AGC) target 3 x 106 at 70,000 resolution; scan range 350–1500 m/z; Orbitrap full-scan maximum injection time 45ms; MS2 scan AGC target 3.2 x 103 at 17,500 resolution; maximum injection 45 ms; normalized collision energy 27; dynamic exclusion time 30 s; isolation window 2.2 m/z; 10 MS2 scans per full scan.

Gel-based Analysis of Crosslinked Proteins

CCRF-CEM cell lysates (16.7 µL at 3 mg/mL in SDS lysis buffer) or dry pellets of human His-TEV-PDE6D-Avitag protein (1 µg resuspended in 16,7 µL H20) previously photolabelled with PAL with or without an excess of DW0254 were mixed with 5,6 µL XT Sample Buffer, 4X (Bio Rad) containing 2.5% v/v 2-mercaptoethanol and heated at 60°C for 30 min. Proteins were resolved by SDS-PAGE using 4–15% gels and analyzed by in-gel fluorescence scanning using a ChemiDocTM MP Imaging System (Bio Rad) with a green LED light as an excitation source and a BP600/20 nm emission filter. After in-gel fluorescence scanning, gels were stained with the PierceTM PageBlueTM protein staining solution and imaged with the ChemiDocTM MP Imaging System.

LC-MS/MS Analysis of PAL in Methanol

PAL photoprobe (500 fmol/µL in 0.05% TFA and 0.2% ACN in water) was analyzed by nanoLC/MS-MS using an Ultimate 3500 RSLC System (Dionex) couple

to a Q-Exactive Plus mass spectrometer. Twenty µl of diluted photoprobe solution (10 pmol) was loaded onto a C18 precolumn at 4°C (Acclaim™ PepMap™ C18, 100 Å, 5 µm, 300 µm ID x 5 mm) at 20 µl/min in 2 % ACN, 0.05 % TFA. After 3 min of desalting, the precolumn was switched online with the analytical C18 column (Acclaim™ PepMap™ RSLC C18, 100 Å, 2 µm, 75 µm ID x 15 cm) equilibrated in 94 % solvent A (0.2 % FA) and 6 % solvent B (80 % ACN, 0.2 % FA). The C18 column was eluted with a linear gradient from 6 to 55 % of solvent B during 24 min at a flow rate of 300 nL/min. The mass spectrometer was operated in the data-dependent mode. using the following settings: full-scan automatic gain control (AGC) target 3 x 106 at 70,000 resolution; scan range 350–1500 m/z; Orbitrap full-scan maximum injection time 30ms; MS2 scan AGC target 3.2 x 103 at 17,500 resolution; maximum injection 45 ms; normalized collision energy 27; dynamic exclusion time 60 s; isolation window 2 m/z; 10 MS2 scans per full scan.

Quantification and Statistical Analysis

MS Data Processing for Intact Cell-Based Photoaffinity Labelling with PAL Probe

Protein identification and quantification were performed using MaxQuant software (version 1.5.3.8) (Cox and Mann, 2008). One quantitative analysis was performed with the 3 conditions: DMSO; PAL alone; PAL with an excess of DW0254 (PAL+DW0254). Raw files were searched against a Swiss-Prot database (Homo Sapiens, 20,200 sequences;) using the following parameters: a maximum of 2 miss-cleavages were allowed and a minimum peptide length of 7 amino acids were required. Trypsin/P was set as the enzyme. Cysteine carbamidomethylation was defined as fixed modification and oxidized methionine, protein N-terminal acetylation and Gln->pyro-Glu as variable modifications. Protein and PSM (peptide-to-spectrum match) false discovery rate (FDR) were set to 0.01. LFQ (Label Free Quantification) intensities calculated by MaxQuant were used for quantification with a min LFQ ratio

count = 1 and the match between run option was enable for an alignment time window of 20 min and 0.7 min matching time.

Proteins identified as “reverse” and as “contaminants” from MaxQuant analyses were discarded from the final list of identified and quantified proteins. Principal component analysis (PCA) was performed on the resulting quantified proteins (based on LFQ values) to identify the main source of variation in a multivariate dataset. In the resulting protein lists, injection replicate data were concatenated. The proteins quantified in less than 2 out of 3 replicates in at least one condition were removed and missing values were replaced by a constant corresponding to the average of the lower inner fences of the different conditions. Statistical differential analysis was performed using Limma (“Linear Models for Microarray Data”) t-test (Smyth, 2004) and Benjamini-Hochberg false discovery rate correction was applied for multiple testing correction. Proteins with a FDR < 5% and a fold change of at least 2 were selected to be differentially modulated. A protein was considered as a target hit of DW0254 when identified with at least two peptides in at least 2 out of 3 replicates, FC > 2 and adjusted p-values < 0.05 in the two comparisons, PAL/DMSO and PAL/PAL+DW0254.

MS Data Processing for In-vitro Photolabeling of PDE6D with PAL Probe

The raw files were processed with the MaxQuant software for peptide and protein identification and quantification. MS/MS raw files of the digests were searched using the Andromeda search engine against a database containing only the recombinant human His-TEV-PDE6D-Avitag sequence using the following parameters: carbamidomethylation of cysteine was set as fixed modification whereas N-terminal acetylation and methionine oxidation were set as variable modifications. All peptides were required to have a minimum peptide length of seven amino acids and a maximum of two miss cleavages. Specificity for Glu-C cleavage was required allowing cleavage after glutamate and aspartate. The false discovery rate (FDR) for

protein and peptide identifications was set to a maximum of 1%. To validate and transfer identifications across different runs, the “match between runs” option in MaxQuant was enabled with a Match time window of 0.7 min and an alignment time window of 20 min. Unknown modifications were identified by the “dependent peptides” setting implemented in MaxQuant in a standard search (Cox et al., 2011). The algorithm performs an unbiased search for modified peptides that are derived from an already identified peptide. If an unidentified spectrum matches an identified spectrum, the mass shift (corresponding to the modification of the peptide) between the theoretical and the observed precursor mass and the matched sequence will be reported. Modified peptides will be only identified if they are derived from an already identified unmodified peptide with an FDR of 1% and a mass tolerance of 6.5 mDa. Modified peptides were extracted from allPeptides.txt along with the ΔM mass shift between the unmodified “base peptide” and the modified peptide. All amino acids were considered as possible residues for modification. The mass of the modification used to search for probe-modified peptides was +581.3002 m/z for PAL which is the mass for the corresponding probe minus two nitrogen atoms. This modification was set as a variable modification in all MaxQuant searches. In brief, for “dependent peptides” analysis, the “all.peptides.txt” file was loaded and filtered for DP Proteins = “sp|DPE6D|”, DP Mass Difference = 581.3002 +/- 10 ppm and DP Score “>60”. Selected peptides with a DP mass shift corresponding to the photoadduct with a tolerance of 10 ppm and which are only present in the two conditions “PAL” and “PAL+DW0254” and absent in the control “DMSO” were considered as positive hits. Remaining hits were further validated in a manual fashion. MS spectra were visualized with the Xcalibur software to validate the presence of the unmodified and modified peptides. Ideally, the unmodified peptide should be detected in all three conditions whereas the peptide modified with a photoadduct should be detected in the condition “PAL” and to a lesser extent in the condition “PAL+DW0254” but not in the control “DMS0”. MS2 spectra were visualized using the viewer program of

MaxQuant to annotate y and b ions of the unmodified peptide. MS2 spectra of the unmodified and modified peptides of interest were analyzed using XCalibur to determine the position of the photo adduct in the sequence. A mass shift corresponding to the photo adduct on a y and/or a b ion is expected.

Computational Analysis of PDE6D mutants and PAL docking

All computational analysis was carried out using MOE. Homology models of the PDE6D R48delV49del reported in Figure 5E were built by using the apo structure of PDE6D as template. Default homology modelling settings in MOE were used. Ten homology models per mutants were built and the best scoring model according to the GB/VI function was selected. Induced fit docking of DW0254 and Deltarasin were carried out. The bioactive conformation of the two ligands was extracted from the respective co-crystallized structure with PDE6D, while positioning in terms of translation and rotation was randomized. The binding site was identified by the superposed 3D coordinates of DW0254. The triangle matcher placement method was used to generate 1000 initial poses disabling the conformational search of the ligand as the bioactive conformation was already available. The generated poses were scored with the London dG function, the 30 best scoring poses were submitted to the induced fit refinement stage allowing residues within 6 Å of the ligand to move. The refined complexes were scored with the GBVI/WSA dg function, and the best scoring one was finally selected. Usually, an RMSD of the docked ligands to crystallographic coordinates lower than 2 Å is considered a successful docking

An initial docking pose for PAL was obtained by manually extending DW0254 with the linker attached to D37. The same energy relaxation protocol described above including minimizations and MD simulations was applied to refine the complex.

Bioluminescent imaging for DW0254 *ex vivo* efficacy studies

To generate a cell line with luciferase expression, P12-ICHIKAWA cells were infected with Lenti-FUW-Luc-mCh-puro virus and selected in liquid culture with puromycin (Sigma-Aldrich) 2.5µg/mL for 7 days following mCherry$^+$ cell sorting.

All animal studies were approved by the Boston Children's Hospital or Dana-Farber Cancer Institute Animal Care and Use Committee. 8- to 10-week-old NOD-scid IL2Rgamma^{null} (NSG) mice (Jackson laboratories, Bar Harbor, ME) were sublethally irradiated with 300 cGy and injected with $1x10^6$ luciferase expressing P12-ICHIKAWA cells treated for 12 hours with DMSO or 3µM DW0254. Disease burden was assessed using bioluminescence imaging starting six days after injections. Prior to imaging, each mouse was given an intra-peritoneal (i.p.) injection of luciferin (PerkinElmer, Part Number #122799) at a dose of 150mg/kg body weight. General anesthesia was then induced with 5% isoflurane and the mouse was placed in the light-tight heated chamber; anesthesia was continued during the procedure with 2% isoflurane introduced via nose cone. Both prone and supine images were recorded.

Optical images were displayed and analyzed with the Igor (WaveMetrics, Lake Oswego, OR) and IVIS Living Image (Xenogen) software packages. Regions were manually drawn around the bodies of the mice to assess signal intensity emitted. Optical signal was expressed as photon flux, in units of photons/s/cm^2/steradian. The total photon flux for each mouse was calculated as the sum of prone and supine photon flux.
